# piR-823 inhibits cell apoptosis via modulating mitophagy by binding to PINK1 in colorectal cancer

**DOI:** 10.1038/s41419-022-04922-6

**Published:** 2022-05-17

**Authors:** Shuling Wang, Xiaoyu Jiang, Xiaoli Xie, Jie Yin, Jiuna Zhang, Ting Liu, Shujia Chen, Yijun Wang, Xue Zhou, Yongjuan Wang, Ruolin Cui, Huiqing Jiang

**Affiliations:** 1 Department of Gastroenterology, The Second Hospital of Hebei Medical University, Hebei Key Laboratory of Gastroenterology, Hebei Institute of Gastroenterology, Hebei Clinical Research Center for Digestive Diseases, Shijiazhuang, Hebei China; 2 Department of Gastroenterology, Shijiazhuang People’s Hospital, Shijiazhuang, 050000 Hebei, China

**Keywords:** Colorectal cancer, Gastrointestinal cancer

## Abstract

Mitophagy plays a vital role in the maintenance of mitochondrial homeostasis and tumorigenesis. Noncoding RNA piR-823 contributes to colorectal tumorigenesis. In this study, we aim to evaluate piR-823-mediated mitophagy and its mechanistic association with colorectal cancer (CRC). Digital gene expression analysis was performed to explore the potential functions of piR-823. A piR-823 antagomir (Ant-823) was used to inhibit piR-823 expression, and piR-823 mimics (mimics-823) were used to increase piR-823 expression. Mitophagy was measured in vivo and in vitro by immunofluorescence and western blot analysis. JC-1 staining, ATP production, real-time PCR, and western blot analysis were used to measure changes in mitochondrial quality and number. siRNA transfection was used to inhibit mitophagy, and CCCP was used to induce mitophagy. RNA pull-down assays and RNA-binding protein immunoprecipitation assays were conducted to investigate the molecular mechanisms. Here, we found that CRC cells transfected with Ant-823 presented an altered expression of autophagic and mitophagy genes by Digital gene expression analysis. Ant-823 could promote Parkin activation and mitophagy in vitro and in vivo, followed by mitochondrial loss and dysfunction of some mitochondria, whereas mimics-823 exerted the opposite effects in CRC cells. The inhibition of mitophagy by siParkin alleviated Ant-823-induced mitochondrial loss and dysfunction, as well as apoptosis to a certain extent. Furthermore, piR-823 was found to interact with PINK1 and promote its ubiquitination and proteasome-dependent degradation, thus alleviating mitophagy. Finally, these findings were verifed in samples obtained by patients affected by colorectal cancer. In conclusion, we identify a novel mechanism by which piR-823 regulates mitophagy during CRC tumorigenesis by increasing PINK1 degradation.

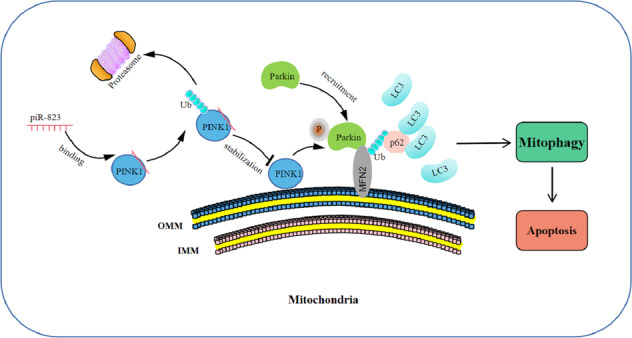

## Introduction

Colorectal cancer (CRC) is the third most prevalent cancer and the second leading cause of cancer-related death worldwide [1]. An increase in the incidence of CRC is likely to result from demographic changes (population growth and aging) and increasingly unfavorable trends in the major risk factors of CRC (physical inactivity, obesity and the adoption of a more Western lifestyle) [2]. Although early prevention and detection have been increasingly applied, large numbers of patients are still diagnosed at the late stages of CRC, resulting in poor prognosis [3]. Therefore, further insight into the molecular mechanisms underlying CRC is important to search for novel therapeutic targets and improve therapeutic efficiency.

Over the past decades, the pivotal role of ncRNAs in regulating carcinogenesis has been shown in several studies [4–6]. Piwi-interacting RNAs (piRNAs) are single-stranded (23–36 nt) ncRNAs with 2’-O-methylated 3’-end modifications. Recent studies have found that piRNAs are expressed in human somatic tissues and involved in the carcinogenesis of different types of tumors. The expression of one such piRNAs, piR-823, is upregulated in multiple myeloma, esophageal squamous cell carcinoma, colorectal cancer, prostate cancer, and breast cancer but downregulated in gastric cancer and renal cell carcinoma [7–12].

Opening of the mitochondrial permeability transition pore (mPTP) is promoted by intracellular stress and decreases the mitochondrial membrane potential, which consequently leads to mitophagy. Meanwhile, the loss of mitochondrial membrane potential precedes caspase-9 and mitochondrial apoptotic pathway activation [13]. Moreover, other studies have shown that aberrant mitophagy disrupts mitochondrial homeostasis, leading to apoptotic cell death [14–16]. Therefore, targeting mitophagy might affect the balance between tumorigenesis and cell death. The previous study from our laboratory demonstrated that the inhibition of piR-823 facilitated colorectal cancer cell apoptosis and decreased expression of the mitochondrial chaperonins HSP60 and HSP70 [8], suggesting that piR-823 might regulate the cell apoptosis and mitochondrial quantity. However, whether mitophagy involved in the regulation of piR-823-associated apoptosis in CRC remains unclear.

In this study, we reported that Ant-823 led to the dysregulation of autophagy and mitophagy-related genes. piR-823 could repress PINK1-Parkin-mediated mitophagy to some extent, and contributed to the maintaining of mitochondrial function, number, and dynamics. Moreover, piR-823 was found to interact with PINK1 and promote the proteasome-mediated degradation of PINK1, leading to the inhibition of mitophagy and the apoptosis of HCT116 and DLD-1 cells. These results reveal intrinsic links among piR-823, mitophagy, and apoptosis and provide a novel mechanism by which piR-823 regulates CRC tumorigenesis.

## Materials and methods

### Patients and samples

CRC tissues and matched adjacent tissues were surgically resected from six patients with CRC and were obtained from the Second Hospital of Hebei Medical University, Shijiazhuang, China. The diagnosis and histological Evaluation of CRC were performed by two pathologists. After excision, tissues were immediately placed at −80 °C, or immediately stored in RNAstore Reagent (Tiangen, Beijing, China), or fixed in 4% paraformaldehyde (Shijiazhuang Huawo Kerui Biological Technology Co., Ltd., Hebei Province, China). This study was approved by the Ethics Committee of the Second Hospital of Hebei Medical University (approval letter No. 2017- R028), and informed consent forms were obtained from all patients.

### Cell culture and treatment

The human CRC cell lines HCT116, HT29 and DLD-1 were obtained from the Shanghai Institute of Biochemistry and Cell Biology, Chinese Academy of Sciences (Shanghai, China), and the human normal colonic epithelial cell line FHC was purchased from American Type Culture Collection (ATCC, Manassas, VA, USA). HCT116 and HT29 cells were maintained in McCoy’s 5A medium (Sigma-Aldrich, St. Louis, MO, USA), DLD-1 cells and FHC cells were cultured in RPMI 1640 medium (Gibco BRL, Rockville, MD, USA) and DMEM/F12 medium (HyClone, Logan, UT, USA), respectively, supplemented with 10% FBS (Gibco BRL), 100 U/mL penicillin and 100 μg/mL streptomycin at 37 °C in a 5% CO_2_ humidified incubator_._ To measure the half-life of PINK1, cycloheximide (CHX, 10 μg/ml, Enzo Life Sciences, Farmingdale, NY, USA) was used to block protein synthesis, while MG132 (10 μM, MCE, NJ, USA) was used to inhibit proteasome-mediated degradation in HCT116 cells. CCCP (40 μM, Sigma-Aldrich) was used to activate mitophagy. HCT116, DLD-1, and HT29 cells were authenticated by Viva Cell Biosciences Ltd (Shanghai, China).

### Transfection

A chemically modified antisense piR-823 sequence (Ant-823, 40 nM, GenePharma Tech, Suzhou, China) was used to downregulate piR-823 expression, and chemically modified piR-823 mimics (mimics-823, 100 nM, GenePharma) was used to upregulate piR-823 expression. Meanwhile, a chemically modified nonspecific scrambled RNA sequence was used as a negative control (Ant-NC or mimics-NC). Their transfection was performed with Lipofectamine 2000 (Invitrogen, Carlsbad, CA, USA) following the manufacturer’s instructions and analyzed 3 days later. HCT116 cells were transfected with lentiviral vectors carrying the antisense sequence of piR-823 (LV-sh-piR-823) or nonspecific scrambled RNA sequence (LV-sh-NC) using polybrene. HCT116 and DLD-1 cells were transfected with either siRNA targeting Parkin (siParkin, 12.5 nM, GenePharma Tech) or PINK1 (siPINK1, 12.5 nM, GenePharma Tech) or a nonspecific scrambled RNA sequence (siNC, 12.5 nM, GenePharma Tech) using Lipofectamine RNAiMAX (Invitrogen) according to the protocol and analyzed 3 days later. The RNA sequences above and lentiviral vectors are presented in Supplementary Table S1.

### RNA and DNA isolation and real-time PCR

Total RNA was extracted using a miRcute miRNA Isolation Kit (Tiangen, Beijing, China). Reverse transcription of total RNA was performed with the miScript Plant RT Kit (Qiagen, Hilden, Germany). The synthesized cDNA was amplified with the miScript SYBR Green PCR Kit (Qiagen). The fold change in piR-823 expression was normalized to U6 expression. Total DNA was extracted using a TIANamp Genomic DNA Kit (Tiangen). Changes in mitochondrial DNA (mtDNA) were measured in comparison with nuclear DNA, represented by HBG. The mtDNA was amplified with SuperReal PreMix Plus (Tiangen). The sequences of primers specific for piR-823, U6, mtDNA, and HGB are shown in Supplementary Table S2. Relative expression was calculated using the 2^-∆∆Ct^ method.

### Western blot analysis and coimmunoprecipitation

HCT116 and DLD-1 cells were lysed in RIPA buffer for total protein extraction and using Mitochondria Isolation Kit (Beyotime, Shanghai, China) for cytoplasmic and mitochondrial protein extraction. The protein concentrations were quantified using a Bradford assay, and proteins were denatured in SDS loading buffer (Solarbio, Beijing, China) by boiling. Equal amounts of the protein-containing supernatant were separately subjected to SDS-PAGE and transferred to a polyvinylidene difluoride membrane. Membranes were blocked with 5% skim milk and incubated with primary antibodies diluted in TBST overnight at 4 °C. After washing with TBST, the membranes were incubated with fluorescence-conjugated secondary antibody (1:10,000, Abcam) for 1 h at room temperature. GAPDH was used as an internal reference. The bands were visualized using an Odyssey infrared imaging system (LI-COR Biosciences), and their intensities were quantified with Image-Pro Plus 6.0 software. The following primary antibodies were used: anti-caspase-9, anti-Parkin, anti-MFN2, anti-LC3B, anti-p62, and anti-COX IV (1:1000, Abways, Shanghai, China); anti-DRP1 (1:500, ABclonal, Wuhan, China); anti-TOM20 (1:2000, Abcam, Cambridge, UK); anti-caspase-3, anti-pS65-Ub, and anti-PARP (1:500, Cell Signaling Technology, Danvers, MA, USA); anti-OPTN, anti-NDP52 and anti-pS65-Parkin and anti-PINK1 (1:500, Affinity Biosciences, Cincinnati, OH, USA).

The lysate samples (100 μg protein) were precleared with protein A/G magnetic beads and then incubated with anti-ubiquitin (Santa Cruz Biotechnology, Santa Cruz, CA, USA) or anti-IgG (as a negative control) at 4 °C for 3 h, followed by incubation with protein A/G magnetic beads overnight. The immune complexes were precipitated with a magnetic stand and separated by SDS-PAGE, followed by western blot analysis.

### Xenograft tumor implantation model

Male BALB/C nude mice aged 4–5 weeks were randomly divided into two groups and subcutaneously implanted with HCT116 cells (0.95 × 10^6^ cells in 0.1 mL of PBS) transfected with LV-sh-piR-823 or Lv-sh-NC (5 mice in each group). Serial measurements of xenograft growth were carried out for 2 weeks, and tumor volume (V) was assessed using the following formula: V = 0.5 × a × b2, where a and b represent the length and width of the tumor, respectively. The current study was approved by the Hebei Medical University Institutional Animal Care and Use Committee (approval letter No. 2021-AE031). All the animals in our study received humane care.

### Immunofluorescence and fluorescence probe labeling technique

Immunofluorescence assays and fluorescence probe labeling technique were performed to show the colocalization of mitochondria and lysosomes in tumor tissue from xenografts, surgically resected tissue samples, and in living HCT116 and DLD-1 cells, respectively. The acquisition of images under a laser scanning confocal microscope (Olympus, Japan).

CRC tissues and mouse xenograft tumors were fixed with 4% paraformaldehyde, embedded in paraffin, and sliced into 4-μm sections. After heat-induced epitope retrieval with 10 mM sodium citrate buffer (pH 6.0; Solarbio), the slices were permeabilized with 0.1% Triton X-100 and blocked with 5% BSA at room temperature. The slices were incubated with primary antibodies overnight at 4 °C. After incubation with secondary antibody for 1 h and DAPI (Solarbio) for 5 min at room temperature, images were captured. The following primary antibodies were used: rabbit anti-TOM20 polyclonal antibody (1:250, Abcam), mouse anti-LAMP1 monoclonal antibody (1:250, BioLegend, San Diego, CA, USA), and mouse anti-p62 (1:200, Abways).

The fusion of mitophagosomes with hydrolase-containing lysosomes in living HCT116 and DLD-1 cells was assessed. Fluorescent probes LysoTracker Red (LTR, Beyotime), (50 nM, 60 min) and MitoTracker Green (MTG, Beyotime), (100 nM, 30 min) were used to stain viable cells for lysosomes and mitochondria, respectively [17]. Hoechst 33342 (APExBIO Technology LLC, Houston, TX, USA), (1 μg/ml, 30 min) was used to mark the nuclei of living cells in confocal dishes. The fresh medium was changed before the acquisition of images.

### Histological and immunohistochemical analyses

CRC tissues and matched adjacent tissues and mouse xenograft tumors were fixed with 4% paraformaldehyde, embedded in paraffin, and sliced into 4-μm sections. Then, the PINK1 (1:100, Affinity Biosciences), pS65-Parkin (1:200, Cell Signaling Technology), and TOM20 (1:200, TOM20) immunohistochemical staining was conducted according to the standard protocols.

### Digital gene expression analysis

Digital gene expression (DGE) tag profiling, GO and KEGG enrichment analysis of differentially expressed genes were performed in HCT116 cells transfected with Ant-823 or Ant-NC and analyzed by the Novogene Bioinformatics Institute (Beijing, China).

### Isolation of the mitochondria-enriched fraction

The separation of mitochondria was conducted by a Mitochondria Isolation Kit (Beyotime). HCT116 and DLD-1 cells were digested, then collected and incubated in Mitochondria Isolation Buffer with PMSF for 15 min on ice. After homogenization, the homogenates were separated into mitochondrial fraction and cytoplasmic proteins by discontinuous density-gradient centrifugation at 600 × *g*, 11,000 × *g,* and 12,000 × *g* at 4 °C.

### JC-1 assays and measurement of ATP production

The mitochondrial membrane potential was measured by JC-1 assay (Beyotime). HCT116 and DLD-1 cells were incubated in a mixture of culture medium and JC-1 working solution in a 1:1 ratio in the dark for 20 min at 37 °C. Then, the cells were washed twice to remove free JC-1. The fresh medium was changed before the acquisition of images under a laser scanning confocal microscope (Olympus, Japan).

The ATP content of the CRC cells was measured using an ATP assay kit (Nanjing Jiancheng Bioengineering Institute, China) by colorimetry of phosphomolybdic acids according to the manufacturer’s instructions.

### Apoptosis assays

HCT116 and DLD-1 cells were transfected with Ant-823 or Ant-NC in combination with Parkin siRNA (siParkin) or negative control (siNC) for 72 h. The cells were then harvested, washed twice with precooled PBS, and assessed with the Annexin V Apoptosis Detection Kit APC by flow cytometry (Thermo Fisher Scientific, Waltham, MA, USA). Cell pellets were resuspended in 100 μL of 1× binding buffer with 5 μL of Annexin V and 5 μL of propidium iodide (PI) and incubated for 15 min at room temperature in the dark. The samples were analyzed by a FACScan flow cytometer (Becton Dickinson, San Jose, United States) after the addition of another 400 μL of binding buffer. The percentage of Annexin V-positive cells was recorded as a measurement of cell apoptosis.

In addition, a One Step TUNEL Apoptosis Assay Kit (Beyotime) was employed to detect apoptosis following the instructions. The apoptotic index is presented as the percentage of TUNEL-positive cells among the total cells.

### Biotinylated RNA pull-down assay

A single desthiobiotinylated nucleotide was attached to the 3’ terminus of the piR-823 mimics and mimics control following the manufacturer’s protocol to directly enrich RNA-binding proteins (RBPs) (or complexes) [18]. The desthiobiotinylated target RNA was bound to streptavidin beads (Thermo Fisher Scientific) before the addition of cell lysate [19]. The beads were washed by adding the appropriate buffer, vortexing, and separating on a magnetic stand. Samples were eluted using non-denaturing Biotin Elution Buffer, and heated with 1× SDS-PAGE loading buffer. The complexes of recruited RNA-protein were evaluated by western blot analysis.

### RNA-binding protein immunoprecipitation (RIP) assay

RIP assays were conducted using the Magna RIP Kit (Millipore, Billerica, MA, USA) [20, 21]. HCT116 cells (2 × 10^7^/sample) were homogenized with RIP Lysis Buffer, followed by incubation with protein A/G magnetic beads coated with rabbit anti-PINK1 (Affinity) or rabbit anti-IgG (Millipore) overnight at 4 °C. The beads were washed with RIP Wash Buffer six times and incubated with proteinase K buffer to digest proteins. RNAs bound to PINK1 were extracted, purified, and analyzed by real-time PCR.

### Statistical analysis

All statistical analyses and graphics were performed using SPSS 16.0 and GraphPad Prism 5 software. Differences between the two groups were analyzed with the Student’s *t* test. For differences among multiple groups, two-way ANOVA was performed. A value of *P* < 0.05 was used to indicate statistical significance.

## Results

### piR-823 Inhibition resulted in the dysregulation of mitophagy-related genes

**Fig. 1:**
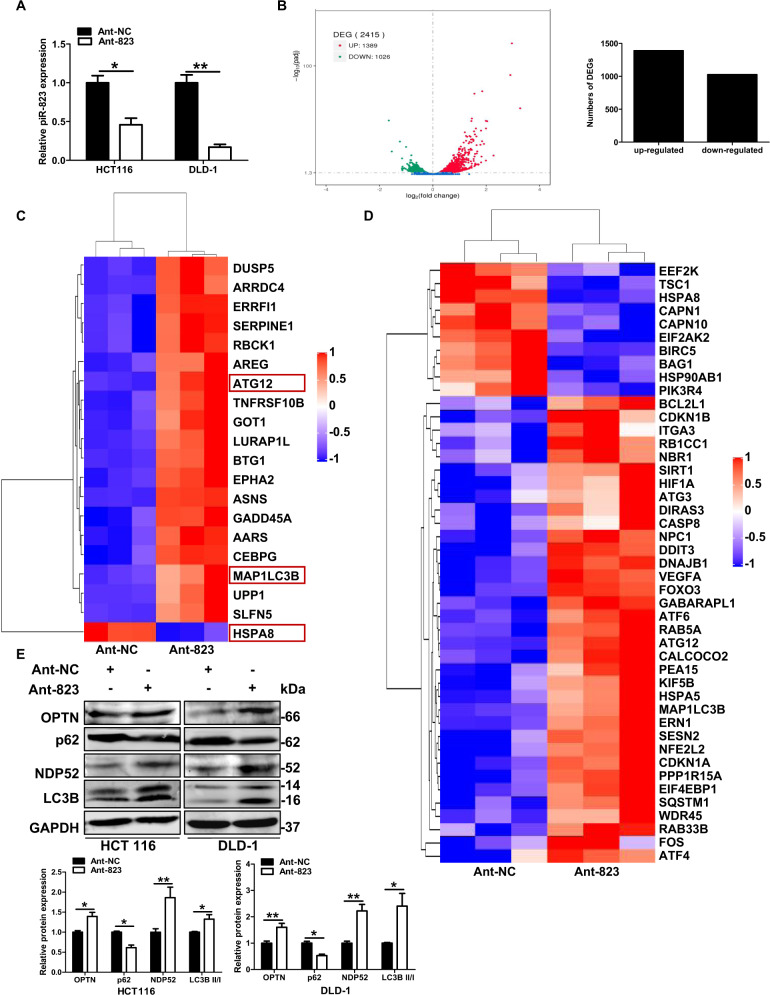
DGE analysis of colorectal cells transfected with Ant-NC and Ant-823. **A** The relative piR-823 level of human CRC cell lines HCT116 and DLD-1 transfected with Ant-NC or Ant-823 by real-time PCR. **B** Differentially expressed genes (DEGs) were detected in different groups (*P* < 0.05). The red part, green part, and blue part represented the upregulated genes, downregulated genes, and genes without expression difference respectively in Ant-823 group compared to control group. **C** The top 20 genes that were most dysregulated by piRNA-823 antagomir treatment. **D** The differentially expressed genes mapped to the autophagy database. **E** The expression changes of mitophagy and autophagy-related proteins after inhibition of piR-823. *P < 0.05, **P < 0.01 vs control groups.

To investigate the potential roles of piR-823 in CRC cells, an antagomir of piR-823 (Ant-823) was used to inhibit piR-823 in HCT116 and DLD-1 cells, and piR-823 mimics (mimics-823) was used to overexpress piR-823 expression in HCT116, DLD-1, HT29, and FHC cells. The transfection efficiency of piR-823 was confirmed by real-time PCR (Fig. 1A for piR-823 inhibition, Supplementary Fig. S1A for piR-823 overexpression). To further explore the molecular mechanism of piR-823 in CRC cells, DGE analysis was performed in HCT116 cells treated with Ant-NC and Ant-823. A total of 2 415 differentially expressed genes were detected. Among these genes, 1 389 (57.52%) genes were upregulated in Ant-823-transfected cells compared to Ant-NC-transfected cells, and 1 026 (42.48%) genes were downregulated (Fig. 1B). Interestingly, among the top 20 genes, three genes (MAP1LC3B/LC3B, ATG12, and HSPA8) were found to be associated with autophagy (Fig. 1C and Supplementary Table S3). Furthermore, when further analyzed, 45 of the differentially expressed genes were mapped to the autophagy database (Fig. 1D), indicating that autophagy might be related to piRNA-823. Moreover, a previous study from our laboratory showed that piR-823 inhibition reduced expression of the mitochondrial chaperonins HSP60 and 70 [8]. Hence, piR-823 is connected with mitochondrial number which was controlled by mitophagy, the selective degradation of mitochondria by autophagy. Further analysis showed that 12 differentially expressed genes were mapped to the mitophagy pathway in the KEGG database. Among these genes, TAX1BP1, SQSTM1/p62, CALCOCO2/NDP52, OPTN, and NBR1 encoding proteins are sequestosome-1-like receptors (SLRs) in PINK1-Parkin-mediated mitophagy in mammals (Supplementary Table S4). Next, we confirmed the expression changes of SQSTM1/p62, CALCOCO2/NDP52, OPTN and LC3B (Fig. 1E for piR-823 inhibition, Supplementary Fig. S1B for piR-823 overexpression). Moreover, all differentially expressed genes were mapped to terms in the GO database. Among the biological process components identified by GO analysis, 57 genes were mapped to the positive regulation of protein serine/threonine kinase activity. On the other hand, among the molecular function components, 61 genes were mapped to the function of ubiquitin-protein ligase binding (Supplementary Table S5). The serine/threonine kinase PINK1 and E3 ubiquitin ligase Parkin play major roles in mitophagy. Based on the above data, we proposed that piR-823 might be involved in the regulation of mitophagy.

### piR-823 repressed PINK1-Parkin-mediated mitophagy

**Fig. 2:**
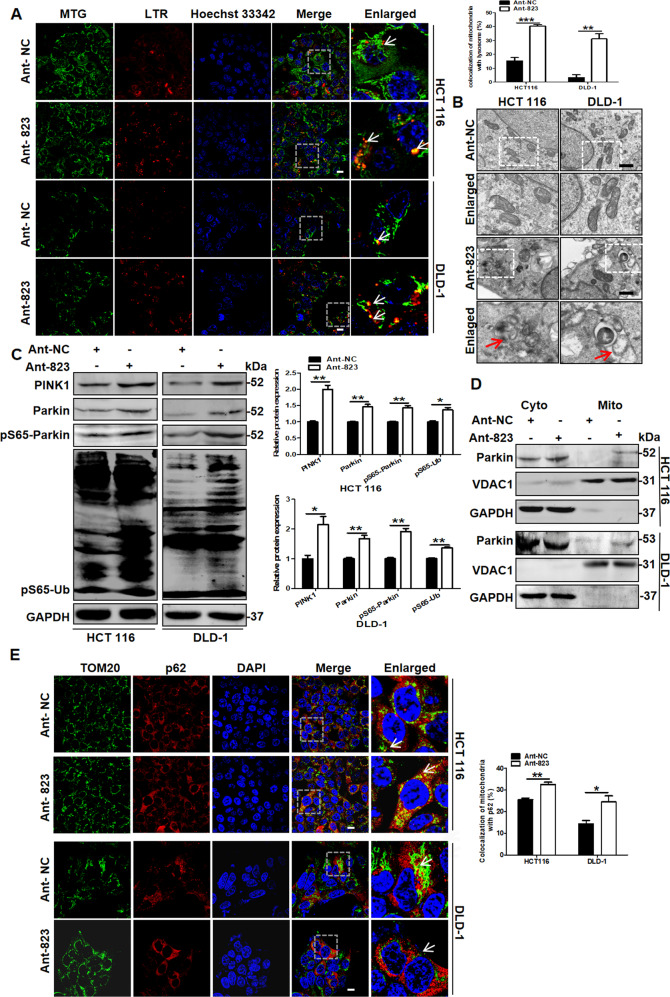
The inhibition of piR-823 facilitates PINK1-Parkin-mediated mitophagy. **A** The colocalization of mitochondria and lysosomes stained with MitoTracker Green (MTG) and LysoTracker Red(LTR) was estimated in HCT116 and DLD-1 cell transfected with Ant-NC or Ant-823. Scale bar: 10 μm. **B** Formation of mitolysosomes was also observed by transmission electron microscopy imaging after piR-823 repression. Scale bar: 1 μm. **C**, **D** Western blot analysis of mitophagy-related proteins and expression of Parkin in the cytosolic and mitochondrial fractions of HCT116 and DLD-1 cells transfeced with the Ant-823 or Ant-NC for 72 h. **E** Mitochondrial colocation with p62 was detected by immunofluorescence. Scale bar : 10 μm. *P < 0.05, **P < 0.01 vs control groups.

To further verify the hypothesis above, we investigated whether piR-823 regulates mitophagy by detecting the colocalization of mitochondria and lysosomes in HCT116 and DLD-1 cells. The inhibition of piR-823 promoted the merged fluorescent signaling of MTG with LTR in the cytoplasm relative to the control group, suggesting the increased colocalization of mitochondria with lysosomes and activation of mitophagy (Fig. 2A), while piR-823 overexpression alleviated the colocalization of mitochondria with lysosomes and activation of mitophagy (Supplementary Fig. S2A). Consistently, the formation of mitolysosomes was also observed in HCT116 and DLD-1 cells transfected with Ant-823 through transmission electron microscopy imaging (Fig. 2B), indicating the activation of Ant-823-induced mitophagy. PINK1-Parkin-mediated mitophagy is a classic mitophagy pathway in which the accumulation of PINK1 at OMM in response to mitochondrial depolarization and the recruitment of Parkin from the cytoplasm to mitochondria are hallmarks of mitophagy [22]. The phosphorylation of Parkin and ubiquitin at Ser 65 by PINK1 is required to activate Parkin E3 ubiquitin ligase activity [23]. Antibodies against phosphorylated ubiquitin (pS65-Ub) have very recently been described as a novel tool to detect the activation of PINK1-Parkin-mediated mitophagy [24]. As shown in this study, repression of piR-823 by Ant-823 facilitated the phosphorylation of Parkin and ubiquitin at Ser 65 in HCT116 and DLD-1 cells and also increased the expression of PINK1 and Parkin, indicating that mitophagy had been partially promoted (Fig. 2C), whereas piR-823 overexpression exerted the opposite effects in HCT116 and DLD-1 cells (Supplementary Fig. S2B). In the cells with piR-823 low expression, such as cancerous HT29 cells and noncancerous FHC cells, introduction of mimics-823 demonstrated similar results (Supplementary Fig. S2C). In addition, enriched Parkin in the mitochondrial fraction was observed after piR-823 inhibition in HCT116 and DLD-1 cells, suggesting the translocation of Parkin from the cytoplasm to mitochondria (Fig. 2D). Parkin ubiquitinates several mitochondrial proteins, such as TOM20, mitofusins (Mfn) and VDAC family members, to help the recognition of damaged mitochondria [25, 26]. These ubiquitylated mitochondrial proteins are targeted by p62/SQSTM1 and bind to LC3 to form the autophagosomes that act in mitophagy [27]. Then, we investigated the roles of p62/SQSTM1 in the Ant-823-indued-mitophagy, and the results showed that p62/SQSTM1 colocalizated with mitochondria in HCT116 and DLD-1 cells transfected with Ant-823, suggesting the mitochondrial anchoring of the autophagy adapter p62/SQSTM1 (Fig. 2E). Altogether, these results indicate that the piR-823 inhibits PINK1-Parkin-mediated mitophagy to a certain extent in vitro.

### piR-823 contributed to the maintaining of mitochondrial function, dynamics, and quantity

**Fig. 3:**
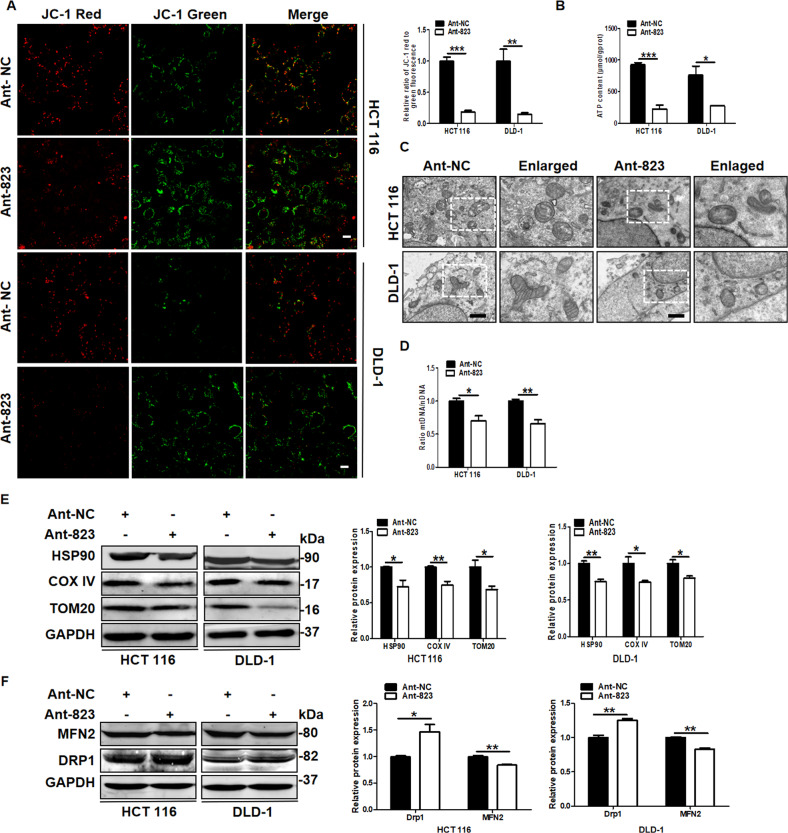
Impaired mitochondrial function, dynamics, and number are observed in HCT116 and DLD-1 cells transfected with Ant-823. **A** JC-1 staining was conducted to evaluate the mitochondrial membrane potential, reflecting mitochondrial function. Scale bar: 10 μm. **B** ATP production was used to reflect the function of mitochondria. **C** Representative images of transmission electron microscope to reveal mitochondrial morphological changes. **D** Real-time PCR analysis of mitochondrial DNA copies. **E** Western blot analysis of HSP90, COX IV and TOM20 were detected to evaluate the mitochondrial quantity. **F** Changes of mitochondrial dynamics in control and piR-823-downregulated-HCT116 and DLD-1 cells. *P < 0.05, **P < 0.01, ***P < 0.001 vs control groups.

Mitophagy is the main process by which poorly functioning or damaged mitochondria are eliminated and thus plays a vital role in the mitochondrial quantity and quality control [28]. Since piR-823 inhibits mitophagy, we next evaluated its impacts on mitochondrial. Compared with the negative control, Ant-823 transfection led to decreased JC-1 red staining but increased JC-1 green staining (Fig. 3A), indicating a decline in mitochondrial membrane potential, and that the piR-823 overexpression had opposite effects in HCT116 and DLD-1 cells (Supplementary Fig. S3A). In addition, the ATP content was significantly decreased after piR-823 inhibition (Fig. 3B) in HCT116 and DLD-1 cells. These data suggest that the inhibition of piR-823 results in aberrant mitochondrial function. Mitochondrial morphological changes observed by transmission electron microscopy showed vague mitochondrial cristae and decreased density of matrix in Ant-823-transfected HCT116 and DLD-1 cells (Fig. 3C). In addition to mitochondrial functional and morphological alteration, mitochondrial number was detected as well. Compared with the control group, the Ant-823 group showed a significantly decreased mtDNA copy number (Fig. 3D), and that mimics-823 group showed a significantly increased mtDNA copy number (Supplementary Fig. S3B), which was quantified to address changes in the relative mitochondrial number. Consistently, downregulating piR-823 decreased the expression of mitochondrial components HSP90, COX IV, and TOM20 (Fig. 3E), whereas they were promoted in cells transfected with mimics-823 (Supplementary Fig. S3C, S3D). Since fusion/fission dynamics is crucial in maintaining of mitochondrial function, we investigated the roles of piR-823 in mitochondrial dynamics. piR-823 inhibition led to a decrease in MFN2 expression but indued an increase in DRP1 expression (Fig. 3F), while piR-823 overexpression showed opposite effects (Supplementary Fig. S3E), suggesting the alteration of fusion/fission dynamics. Altogether, these results demonstrate that piR-823 repression can impair mitochondrial function and dynamics, and leads to mitochondrial loss in CRC cells.

### piR-823 inhibition induced mitochondrial dysfunction and loss by provoking mitophagy

**Fig. 4:**
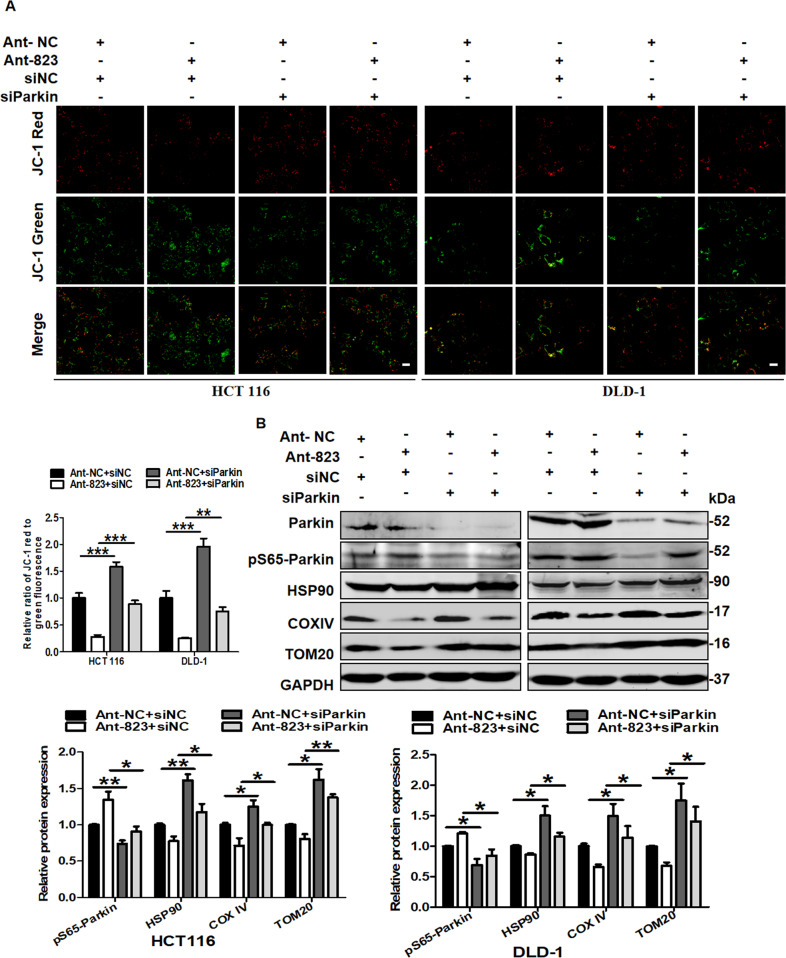
Inhibition of mitophagy alleviated piR-823 inhibition-induced mitochondrial dysfunction and loss. **A** Mitochondrial potential changes detected via JC-1 staining. **B** Western blot analysis of Parkin, pS65-Parkin, HSP90, COX IV, and TOM20 in HCT116 and DLD-1 cells transfected with siNC or siParkin in combination with Ant-NC or Ant-823 for 72 h. *P < 0.05, **P < 0.01, ***P < 0.001 *vs* control groups.

Mitophagy is an important event in the control of mitochondrial quality and quantity. We then explored whether mitophagy inhibition mediated by siParkin would relieve Ant-823-induced mitochondrial dysfunction and loss. Compared with the Ant-823 and siNC treatment groups, the Ant-823 plus siParkin group showed increased JC-1 red staining but decreased JC-1 green staining, indicating an increase in the mitochondrial membrane potential (Fig. 4A). Consistently, the addition of siParkin alleviated the expression levels of pS65-Parkin and partially restored the mitochondrial number, as evidenced by increased HSP90, COX IV, and TOM20 expression levels (Fig. 4B). In contrast, upon CCCP treatment to activate mitophagy, mimics-823 showed the important regulations in the maintaining of mitochondrial function and number (Supplementary Fig. S4A, B). Thus, these data suggest that mitochondrial dysfunction and loss induced by inhibition of piR-823 might attribute to the activation of mitophagy.

### Inhibition of mitophagy alleviated piR-823 inhibition-induced apoptosis

**Fig. 5:**
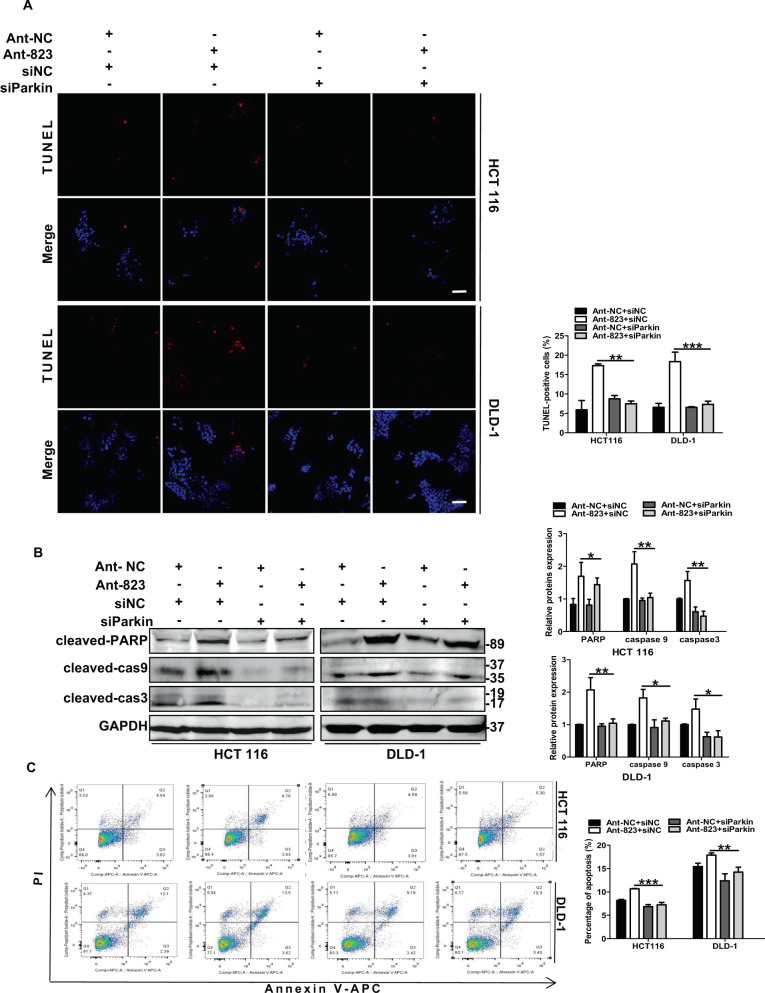
Mitophagy induced by the inhibition of piR-823 potentiates apoptotic cell death in CRC cells. **A** TUNEL Apoptosis Assay was employed to analyze apoptosis. Scale bar: 10 μm. **B** Western blot analysis of cleaved PARP, cleaved-caspase-9 and cleaved-caspase-3. **C** Flow cytometric analysis of apoptosis in HCT116 and DLD-1 cells transtected with siNC or siParkin in combination with Ant-NC or Ant-823 for 72 h. Data are mean ± SD from at least three independent experiments, *P < 0.05, **P < 0.01, ***P < 0.001 vs control groups.

**Fig. 6:**
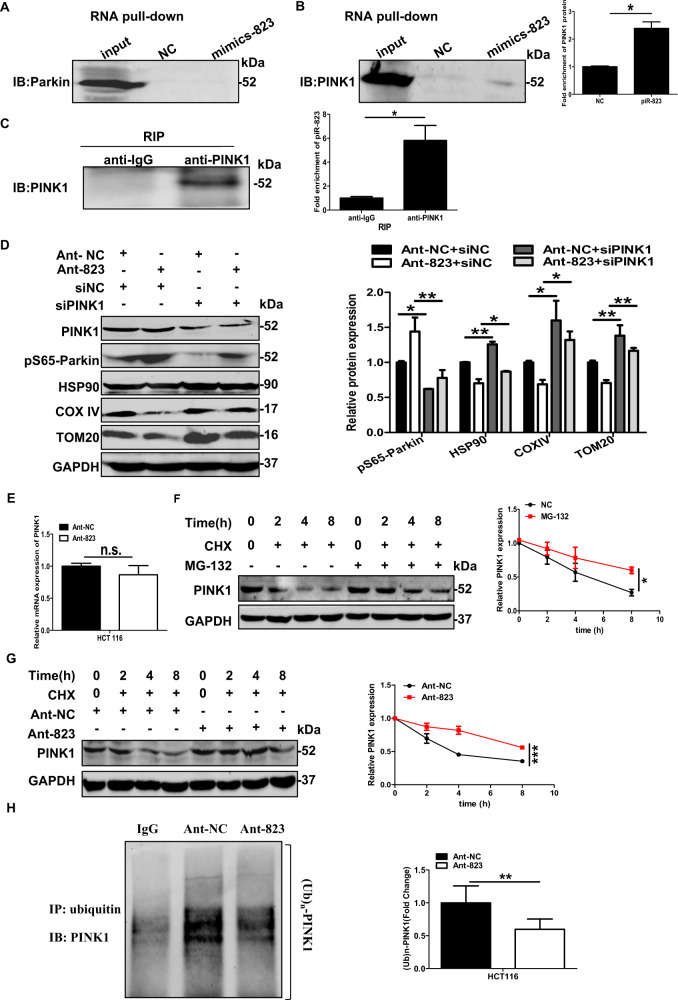
piR-823 interacts with PINK1 and facilitates the ubiquitination and proteasome-dependent degradation of PNIK1 in HCT116 cells. **A**, **B** RNA-binding proteins resulted from RNA-protein pull-down was separated and incubated with anti-Parkin and anti-PINK1. **C** Real-time PCR to detect the level of piR-823 bound to PINK1. **D** Western blot analysis of PINK1, pS65-Parkin, HSP90, COX IV, and TOM20 in HCT116 cells transfected with siNC or siPINK1 in combination with Ant-NC or Ant-823. **E** The mRNA levels of PINK1 in HCT116 cells transfected with Ant-823 or Ant-NC. **F**, **G** Western blot analysis for expression of PINK1 in HCT116 cells transfected with Ant-NC or Ant-823 or in combination with MG132. **H** Immunoprecipitation for PINK1 ubiquitination in HCT116 cells. *P < 0.05 vs control groups; n.s. indicates not significant.

As mitophagy can lead to cell apoptosis, piR-823 inhibits apoptosis in HCT116 and DLD-1 cells, we next addressed whether Ant-823-induced mitophagy is involved in apoptotic cell death. The inhibition of Ant-823-induced mitophagy through siParkin partly rescued the Ant-823-dependent increase in apoptosis in CRC cells, as evidenced by a decreased proportion of TUNEL-positive cells (Fig. 5A). Moreover, the introduction of Parkin siRNA led to a decrease in the protein levels of cleaved PARP, cleaved-caspase-9, and cleaved-caspase-3 in piR-823-inhibited cells relative to the control group (Fig. 6B). Similarly, Ant-823 plus Parkin siRNA rescued the increase in apoptosis induced by Ant-823 alone, as evidenced by the decreased percentage of apoptotic cells (Annexin V-positive cells) among HCT116 cells and DLD-1 cells (Fig. 6C). Collectively, these data demonstrate that stimulated mitophagy partially provokes Ant-823-dependent apoptosis in CRC cells. Contrarily, introduction of mimics-823 alleviated apoptosis, and the treatment of CCCP to provoke mitophagy facilitated apoptotic cell death in HCT116 and DLD-1 cells (Supplementary Fig. S4C).

### piR-823 interacted with PINK1 and facilitated its proteasome-mediated degradation

By DGE analysis, piR-823 inhibition was related to the positive regulation of protein serine/threonine kinase activity and ubiquitin-protein ligase binding. The serine/threonine kinase PINK1 and E3 ubiquitin ligase Parkin play major roles in mitophagy [29]. To investigate the mechanism of how piR-823 works, the protein mixture captured by piR-823 RNA through RNA pull-down assay was validated by western blot analysis and incubated with anti-PINK1 and anti-Parkin. The results indicated that PINK1 was within the piR-823 RNA probe pull-down samples but not Parkin (Fig. 6A, B). The RIP assay affirmed the association between piR-823 and PINK1, as determined by the enrichment of piR-823 detected by real-time PCR by anti-PINK1 but not anti-IgG (Fig. 6C). We next evaluated whether silencing PINK1 would affect the mitophagy induced by Ant-823. Consistently, western blot analysis showed that Ant-823 plus PINK1 siRNA attenuated the elevated expression levels of PINK1 and pS65-Parkin but rescued the decrease in HSP90, COX IV and TOM20 levels induced by Ant-823 alone (Fig. 6D). Taken together, these results indicate that piR-823 might regulate mitophagy through its binding to PINK1.

PINK1 is a serine/threonine kinase that stabilizes selectively at the OMM on damaged mitochondria where it can recruit the E3 ligase Parkin from the cytosol to mitochondria and phosphorylate Parkin and ubiquitin at Ser 65 to induce mitophagy. In this study, piR-823 inhibition increased the expression levels of PINK1. However, there was no significant difference in PINK1 mRNA levels between the HCT116 cells transfected with Ant-823 and Ant-NC (Fig. 6E). These results indicated that the regulation of PINK1 by piR-823 may not in the transcriptional level, and piR-823 might regulate the degradation, but not the production of PINK1. Next, the half-life of the PINK1 protein was examined upon the inhibition of *de novo* PINK1 protein synthesis using CHX. The half-life of PINK1 was extended in the presence of MG132, a proteasome inhibitor (Fig. 6F), suggesting that PINK1 is degraded in a proteasome-dependent manner. Delayed decay of the PINK1 protein was observed after the inhibition of piR-823 (Fig. 6G). Consistently, immunoprecipitation for ubiquitinated PINK1 in HCT116 cells also showed that the inhibition of piR-823 decreased the polyubiquitination of PINK1 (Fig. 6H), whereas overexpression of piR-823 facilitated the polyubiquitination of PINK1 (Supplementary Fig. S5A). Collectively, these data demonstrated that piR-823 promoted the proteasome-mediated degradation of PINK1, thus leading to the inhibition of mitophagy. Concerning that Parkin ubiquitinates several mitochondrial proteins in the activation of mitophagy, we wondered if piR-823 regulate the ubiquitination of mitochondrial proteins. Interestingly, the ubiquitination of TOM20 and MFN2 was facilitated in HCT116 cells transfected with Ant-823, but alleviated in HCT116 cells transfected with mimics-823 (Supplementary Fig. S5B–E), suggesting that piR-823 itself could not regulate the ubiquitination of TOM20 and MFN2 directly, but through the PINK1-Parkin pathway.

### piR-823 inhibition facilitated mitophagy in vivo

**Fig. 7:**
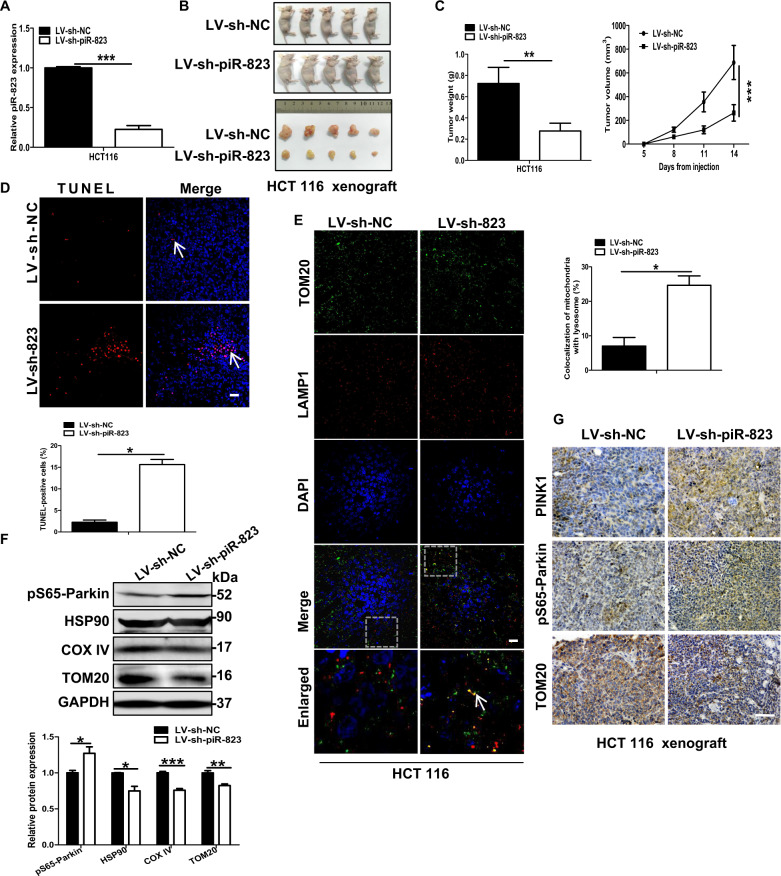
piR-823 inhibition facilitated mitophagy in vivo. **A** The relative piR-823 level of human CRC cell lines HCT116 transfected with LV-sh-NC or LV-sh-piR-823 by real-time PCR. **B** Morphologies of tumors in xenografts tumor model in nude mice (*n* = 5 for each group). **C** Growth curve represented tumor volumes at different time points were shown, as well as the tumor weight of HCT116 xenograft (*n* = 5 for each group). **D** TUNEL Apoptosis Assays were conducted in tumor tissues of HCT116 xenograft (*n* = 5 for each group). Scale bar: 10 μm. **E** Immunofluorescence assays were performed to show the co-location of mitochondria and lysosomes in HCT116 xenografts collected from the control group or piR-823 downregulated nude mice (*n* = 5 for each group). Scale bar: 10 μm. **F** Western blot analysis of pS65-Parkin, HSP90, COX IV and TOM20 were detected to evaluate the mitophagy and mitochondrial quantity in HCT116 xenograft tumors (*n* = 5 for each group). **G** The xenograft tissues were subjected to immunohistochemistry staining for PINK1, pS65-Parkin, and COX IV. Scale bar: 100 μm.

To further explore the in vivo observations, a xenograft model in nude mice was developed with HCT116 cells transfected with the lentiviral vectors LV-sh-piR-823 or LV-sh-NC. The expression level of piR-823 was decreased after LV-sh-piR-823 transfection in HCT116 cells (Fig. 7A). Tumor volume and tumor weight were significantly reduced in the LV-sh-piR-823 group relative to the control group, which demonstrated that inhibition of piR-823 impair tumor growth (Fig. 7B, C). Compaired with the control group, inhibition of piR-823 led to an increase of TUNEL-positive cells in the LV-sh-piR-823 group (Fig. 7D). Moreover, inhibition of piR-823 increased the merged fluorescent signaling for TOM20 and LAMP1 in the LV-sh-piR-823 group, indicating the activation of mitophagy (Fig. 7E). Consistently, elevated pS65-Parkin expression levels but decreased HSP90, COX IV and TOM20 protein expression levels were observed in piR-823-downregulated HCT116 xenograft tissues relative to the controls (Fig. 7F). In line with the above results, elevated expression levels of PINK1 and pS65-Parkin and decreased expression of TOM20 were detected in xenograft tissues from the LV-sh-piR-823 group by immunohistochemical staining relative to control group (Fig. 7G). Taken together, these data indicate that inhibition of piR-823 contributes to the activation of mitophagy and reduction of mitochondrial number in the xenograft model.

### piR-823 regulated mitophagy in CRC samples

**Fig. 8:**
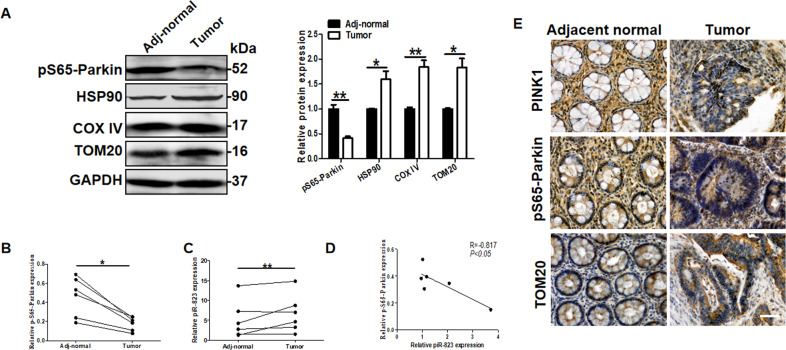
piR-823 regulated mitophagy in CRC samples and matched adjacent tissues. **A, B** Western blot analysis of pS65-Parkin, HSP90, COX IV and TOM20 were detected to evaluate the mitophagy and mitochondrial quantity in CRC tissues and matched adjacent tissues (n = 6 for each group). **C** The relative piR-823 level of CRC tissues and matched adjacent tissues by real-time PCR (n = 6 for each group). **D** Pearson’s correlation and linear analysis. **E** Immunohistochemical staining for PINK1, pS65-Parkin, and TOM20 in CRC tissues and matched adjacent tissues (n = 6 for each group). Scale bar: 100 μm. *P < 0.05, **P < 0.01, ***P < 0.001 vs control groups.

We verified our finding in samples obtained by patients affected by colorectal cancer. Decreased expression levels pS65-Parkin and elevated expression of HSP90, COX IV and TOM20 were detected by western blot analysis, and that increased piR-823 expression levels were detected by real-time PCR in CRC tissues and matched adjacent tissues (Fig. 8A–C). Furthermore, the Pearson correlation and linear regression analyses were performed to explore the relationship between piR-823 and mitophagy, which indicated that piR-823 expression is negatively associated with pS65-Parkin expression (Fig. 8D). Consistently, decreased expression levels of PINK1 and pS65-Parkin and elevated expression of TOM20 were detected in CRC tissues and matched adjacent tissues by immunohistochemical staining (Fig. 8E). Altogether, piR-823 might regulate mitophagy in vivo.

## Discussion

piRNAs are known to interact with PIWI proteins in germ cells and stem cells during early development. However, recent evidence has shown that piRNAs are also expressed in many adult differentiated cell types [30, 31], play different roles in a variety of cancer types, and correlate with tumorigenesis [7–12]. This study showed that CRC cells transfected with Ant-823 presented an altered expression of autophagic and mitophagy genes. Ant-823 facilitated the PINK1-Parkin mitophagy and apoptosis and that by silencing Parkin was possible to reduce these effects. Finally, we demonstrated that piR-823 interacted with PINK1 to determine its proteasome-mediated degradation and reducing the Parkin translation and apoptosis. Overall, we demonstrated that piR-823 regulated mitophagy during CRC tumorigenesis by increasing PINK1 degradation. Our findings provided a novel mechanism by which piR-823 might regulate CRC tumorigenesis and revealed cross-links among piR-823, mitophagy and apoptosis, suggesting that targeting piR-823 may be a new strategy for the treatment of patients with CRC.

Zucchini (Zuc), one of the nucleases required for piRNA biogenesis, processes piRNA molecules from their longer RNA precursors into shorter intermediate forms [32], which subsequently mature to their functional forms by the action of another protein called Trimmer. Interestingly, both Zucchini (Zuc) and Trimmer are nucleases required for piRNA biogenesis [32] and localize to mitochondria [33]. Furthermore, mitochondrial phospholipase D (MitoPLD), the mammalian homolog of Zuc, hydrolyzes the mitochondria-specific lipid cardiolipin, which serves as a recognition signal for dysfunctional mitochondria, thus inducing mitophagy [34]. This makes it possible in space and that piRNAs may be involved in the regulation of mitophagy.

Mitophagy is an important event in the control of mitochondrial quality and quantity. Dysfunctional mitochondria, which would otherwise damage the cell, are engulfed and degraded through the fusion of mitophagosomes with hydrolase-containing lysosomes [29]. Mitochondrial depolarization stabilizes the kinase PINK1 at the outer mitochondrial membrane, which is followed by the phosphorylation of Parkin at Ser 65 homologous to the site where ubiquitin is phosphorylated to activate Parkin, and the subsequent ubiquitination of other proteins and autophagic destruction [26, 29]. In this study, piR-823 contributed to the maintaining of mitochondrial function and quantity by repression of mitophagy, suggesting a regulatory role for piR-823 in mitophagy. Numerous studies on the involvement of another type of noncoding RNA, miRNAs, in mitophagy regulation have been published [35]. However, to the best of our knowledge, this is the first report on the roles of piRNAs in mitophagy.

In addition, mitophagy plays a vital role in maintaining mitochondrial homeostasis and is involved in tumorigenesis and cell death. Mitophagy is generally considered to promote cell survival and inhibit cell apoptosis [36, 37] by degrading dysfunctional mitochondria, which would otherwise release caspase-activating factors [38]. However, several previous studies have shown that aberrant mitophagy disrupts mitochondrial homeostasis, leading to apoptotic cell death [15, 16]. On the other hand, another study demonstrated that the mitochondria-targeted drugs Mito-CP and Mito-Met stimulate mitophagy and abrogate cancer cell proliferation [39]. Ketoconazole-induced mitophagy in hepatocellular carcinoma cells was found to potentiate apoptotic cell death [14]. Similarly, mitophagy induced by melatonin could sensitize HCC cells to apoptosis. Several studies have revealed that PINK1, Parkin, BNIP3, and NIX, the most important mitophagy receptors on the OMM, are tumor suppressor genes involved in promoting apoptosis [29, 40], implying the positive correlation between mitophagy and apoptosis. In line with the above reports, the current study shows that enhanced mitophagy induced after piR-823 inhibition promotes the apoptosis of HCT116 and DLD-1 cells. Furthermore, inhibition of mitophagy by Parkin knockdown restored mitochondrial dysfunction and loss, indicating that the inhibition of piR-823 induces mitochondrial damage by triggering mitophagy in CRC cells and that such antitumor mitophagy is likely to be excessive.

In our previous work, inhibition of piR-823 suppressed cell proliferation, arrested the cell cycle in the G1 phase and induced cell apoptosis in CRC cell lines HCT116 and DLD-1, whereas overexpression of piR-823 promoted cell proliferation in normal colonic epithelial cell line FHC [8]. The HCT116 cells xenograft model in nude mice also showed that piR-823 inhibition impair tumor growth. However, cell lines and xenografts do not faithfully model endogenous Tumors. To verify wtether piR-823 is sufficient to drive the tumorigenesis, a conditional transgenic mouse models of CRC is needed for further research.

Functionally, piRNAs play an epigenetic regulatory role by silencing transposable elements and regulating DNA methylation to maintain genomic integrity [30]. In addition, piRNAs can posttranscriptionally regulate the expression of mRNAs by enhancing the cleavage of mRNAs through an miRNA-like mechanism. To our surprise, in this study, we demonstrated that piR-823 interacts with PINK1, and facilitated the ubiquitination of PINK1, but alleviated the ubiquitination of mitochondrial proteins TOM20 and MFN2. It seems to be a contradiction. However, the ubiquitination of TOM20 and MFN2 is downstream event of stabilization of PNIK1 at the OMM on damaged mitochondria, thus the alleviated ubiquitination of TOM20 and MFN2 is likely to attribute to the repression of PINK1-Parkin-mediated mitophagy induced by Ant-823. In other words, piR-823 regulates the ubiquitination of mitochondrial proteins TOM20 and MFN2 through the PINK-Parkin pathway.

Indeed, previous studies from our laboratory showed that piR-823 increased the transcriptional activity of HSF1 by binding to HSF1 and modulating its phosphorylation [8]. Another study from our laboratory showed that piR-823 bound to EIF3B and increased TGF-β1 expression to activate hepatic stellate cells [41]. These findings indicate that piRNAs may regulate protein modifications by directly interacting with target proteins and modulating their ubiquitination or transcriptional activity.

In summary, this study identifies a novel mechanism by which piR-823 regulates mitophagy during CRC tumorigenesis by increasing PINK1 degradation and Parkin translocation.

## Supplementary information

Supplementary Tables

WB-gel-1

WB-gel-2

WB-gel-3

WB-gel-4

WB-gel-5

WB-gel-6

WB-gel-7

WB-gel-8

WB-gel-9

WB-gel-10

WB-gel-11

Supplementary Fig. 1

Supplementary Fig. 2

Supplementary Fig. 3

Supplementary Fig. 4

Supplementary Fig. 5

Extended Data 1

Extended Data 2

Extended Data 3

Extended Data 4

Extended Data 5

Extended Data 6

## Data Availability

The data that support the findings of this study are available from the corresponding author upon reasonable request.
