# Supplementary material for: piR-823 inhibits cell apoptosis via modulating mitophagy by binding to PINK1 in colorectal cancer
Source: Cell Death Dis. 2022 May 17;13(5):465. doi: 10.1038/s41419-022-04922-6 (PMC9114376; doi:10.1038/s41419-022-04922-6)
Supplement: Supplementary file 1 — Supplementary Tables [file 41419_2022_4922_MOESM1_ESM.docx]

**Supplementary Table S1. Antagomir and siRNA sequences**

|  | **Sense (5'-3')** | **Antisense (5'-3')** |
| --- | --- | --- |
| antagomir-NC (human) | GCAAACCGAGUCCCCUAACGCUUCCCUACUAA | - |
| antagomir-823 (human) | GCAGCUAUGCUCACCACUAUACCACCAACGCU | - |
| mimics-NC | UUCUCCGAACGUGUCACGUTT | ACGUGACACGUUCGGAGAATT |
| mimics-823 | AGCGUUGGUGGUAUAGUGGUGAGCAUAGCUGC | AGCUAUGCUCACCACUAUACCACCAACGCUUU |
| siNC | UUCUCCGAACGUGUCACGUTT | ACGUGACACGUUCGGAGAATT |
| siParkin | GCCACGUGAUUUGCUUAGATT | UCUAAGCAAAUCACGUGGCTT |
| siPINK1 | GGAGCAGUCACUUACAGAATT | UUCUGUAAGUGACUGCUCCTT |

**Supplementary Table S2. Primer sequences**

|  | **Forward primer (5'-3')** | **Reverse primer (5'-3')** |
| --- | --- | --- |
| mtDNA (human) | CACCCAAGAACAGGGTTTGT | TGGCCATGGGTATGTTGTTA |
| HGB (human) | GCTTCTGACACAACTGTGTTCACTAGC | CACCAACTTCATCCACGTTCACC |
| piR-823 (human) | AGCGTTGGTGGTATAGTGGT | - |
| U6 (human) | CTCGCTTCGGCAGCACA | AACGCTTCACGAATTTGCGT |

**Supplementary Table S3. Three genes are associated with autophagy in Ant-823-treated samples by DGE**

| **Gene symbol** | **Description** | ***P* value** |
| --- | --- | --- |
| MAP1LC3B/LC3B | Microtubule associated protein 1 light chain 3 beta | 5.65E-46 |
| ATG12 | Autophagy related 12 | 6.07E-53 |
| HSPA8 | Heat shock protein family A (HSP70) member 8 | 1.53E-53 |

**Supplementary Table S4. Identification of genes mapped to the mitophagy pathway in the KEGG database**

| **Gene symbol** | **Description** | ***P* value** |
| --- | --- | --- |
| MAP1LC3B/LC3B | Microtubule associated protein 1 light chain 3 beta | 5.65E-46 |
| CALCOCO2/NDP52 | Calcium binding and coiled-coil domain 2/ Nuclear dot protein 52 | 1.96E-12 |
| TFE3 | Transcription factor binding to IGHM enhancer 3 | 4.94E-10 |
| SQSTM1/p62 | sequestosome1 | 1.17E-08 |
| TAX1BP1 | Tax1 (human T-cell leukemia virus type I) binding protein 1 | 4.54E-06 |
| HIF1A | Hypoxia inducible factor 1 alpha subunit | 6.65E-06 |
| ATF4 | Activating transcription factor 4 | 2.86E-05 |
| NBR1 | Neighbor of BRCA1 gene1 | 0.000125 |
| TBC1D17 | TBC1domain family member 17 | 0.001494 |
| OPTN | optineurin | 0.006510 |
| USP8 | Ubiquitin specific peptidase 8 | 0.007501 |
| E2F1 | E2F transcription factor 1 | 0.007521 |

**Supplementary Table S5. Identification of differentially expressed genes mapped to GO database**

| **Category** | **Description** | **GeneRatio** | ***P* value** | **Gene ID** |
| --- | --- | --- | --- | --- |
| Biological Process | positive regulation of protein serine/threonine kinase activity | 57/2053 | 0.001601 | DUSP5/GADD45A/TNFRSF10B/GADD45B/AJUBA/EREG/VEGFA/ERN1/PDGFB/SOD1/AVPI1/MAP2K3/MALT1/ADAM17/NEK1/RIPK2/PEA15/FGF2/HSP90AB1/ADORA2B/TAB2/UBC/PIM1/THBS1/CAB39/KIAA1804/STIL/SIRT1/AKT1/AXIN1/CALM2/CALM3/TGFA/PSMD10/HACD3/MDFI/UBB/TAOK3/PXN/DUSP6/TAB1/PSEN1/STK4/SPAG9/KRAS/MAP3K14/FZD4/SHC1/ARRB1/KITLG/MST1R/IRAK2/PTPN1/UBA52/TNFRSF10A/MAP2K5/ARHGEF5 |
| Molecular Function | ubiquitin protein ligase binding | 61/2053 | 4.30E-05 | HSPA8/MAP1LC3B/HSPA1B/CDKN1A/HSPA5/DNAJA1/TUBB/SUMO2/PER1/TRIB3/HSPA9/KDM4A/SQSTM1/VCP/XBP1/EGR2/AXIN2/SMAD3/TRAF3/RFFL/UBE2O/WFS1/TRIM28/SMAD6/BCL10/UBE2B/HIF1A/GABARAPL1/SPOP/SLC22A18/SNX9/SLC25A5/USP25/TUBA1B/UBE2W/BLZF1/PML/AXIN1/RBX1/TRIB2/BRCA1/JAK1/ARIH1/CASP8/ANAPC2/MOAP1/UBE2H/TMEM189/SKI/UBE2J1/FZD4/PDE4D/UBE2S/FZD6/ARRB1/NGFR/ACTG1/EIF4E2/CCDC50/KCNH2/TPI1 |
