# Supplementary figures and images for: piR-823 inhibits cell apoptosis via modulating mitophagy by binding to PINK1 in colorectal cancer
Source: Cell Death Dis. 2022 May 17;13(5):465. doi: 10.1038/s41419-022-04922-6 (PMC9114376; doi:10.1038/s41419-022-04922-6)

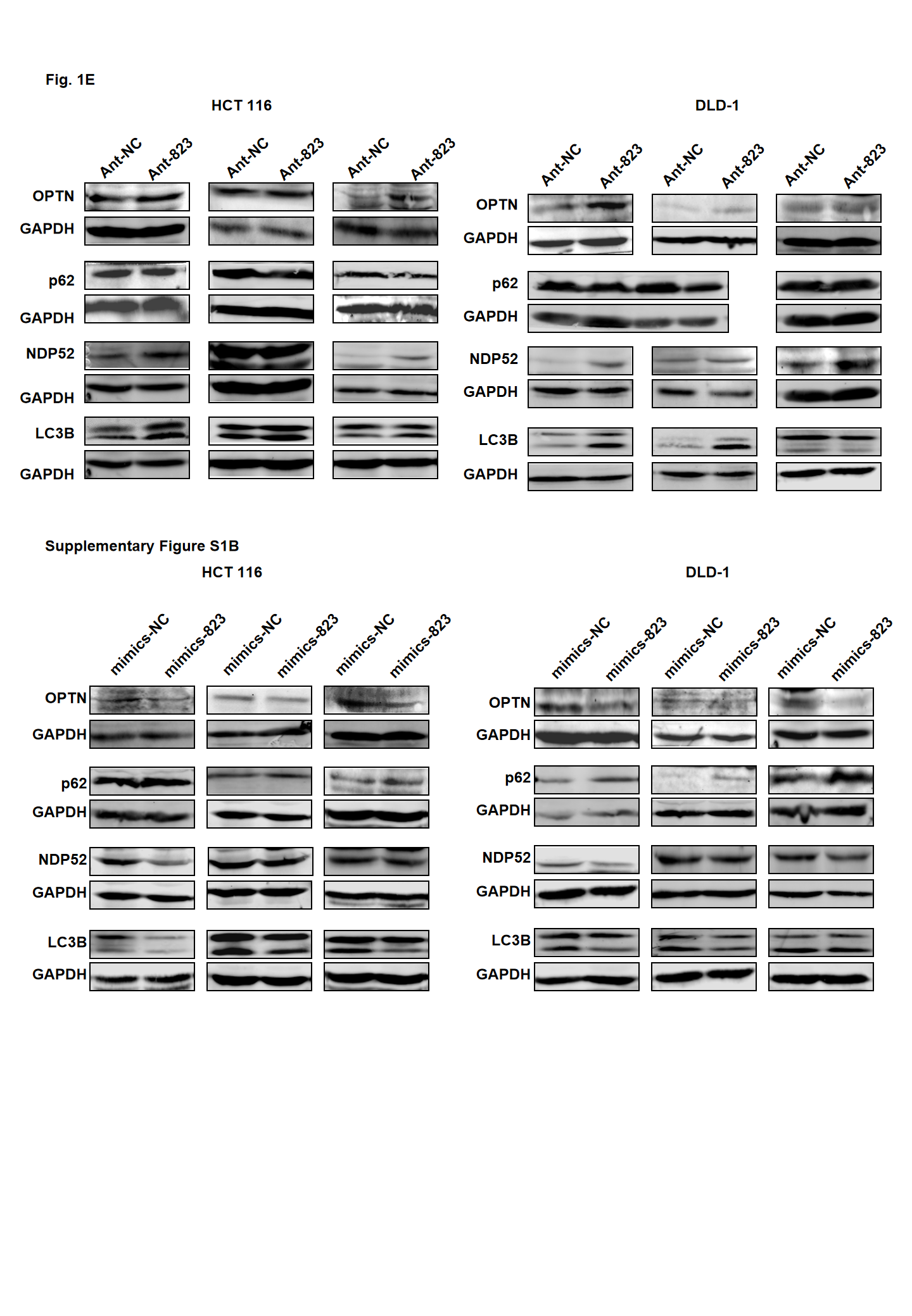

Supplement: Supplementary file 2 — WB-gel-1 [file 41419_2022_4922_MOESM2_ESM.tif]

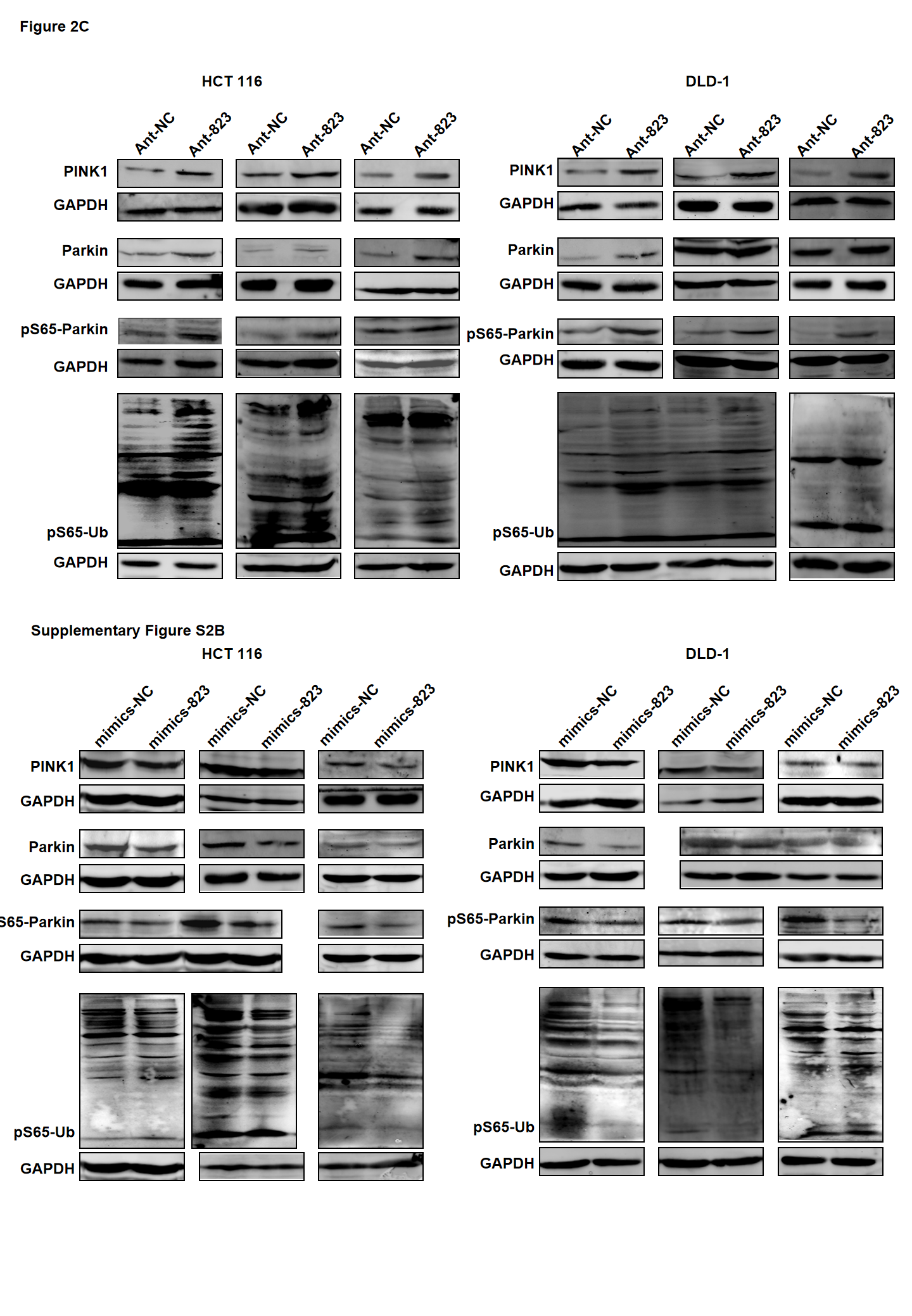

Supplement: Supplementary file 3 — WB-gel-2 [file 41419_2022_4922_MOESM3_ESM.tif]

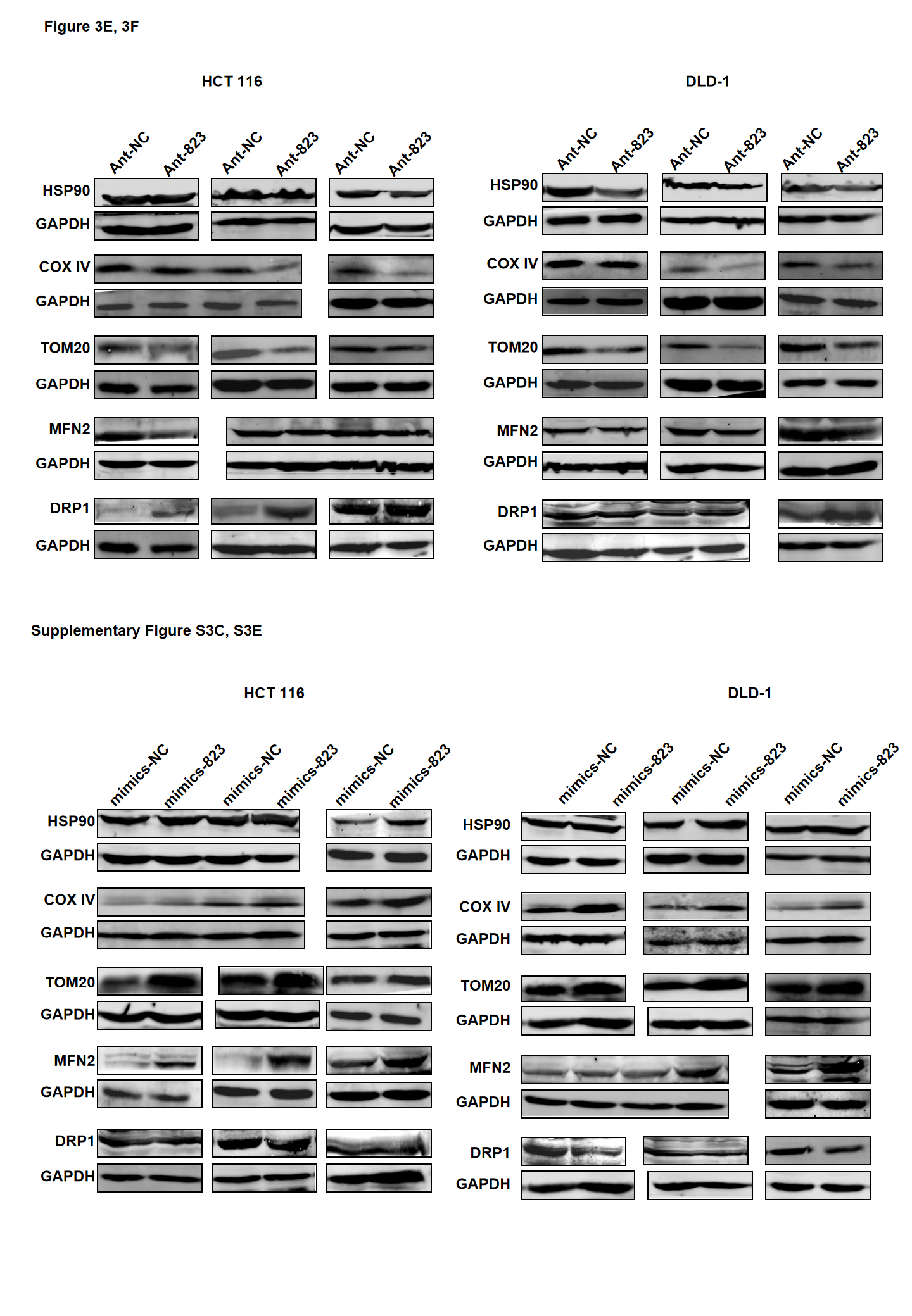

Supplement: Supplementary file 4 — WB-gel-3 [file 41419_2022_4922_MOESM4_ESM.tif]

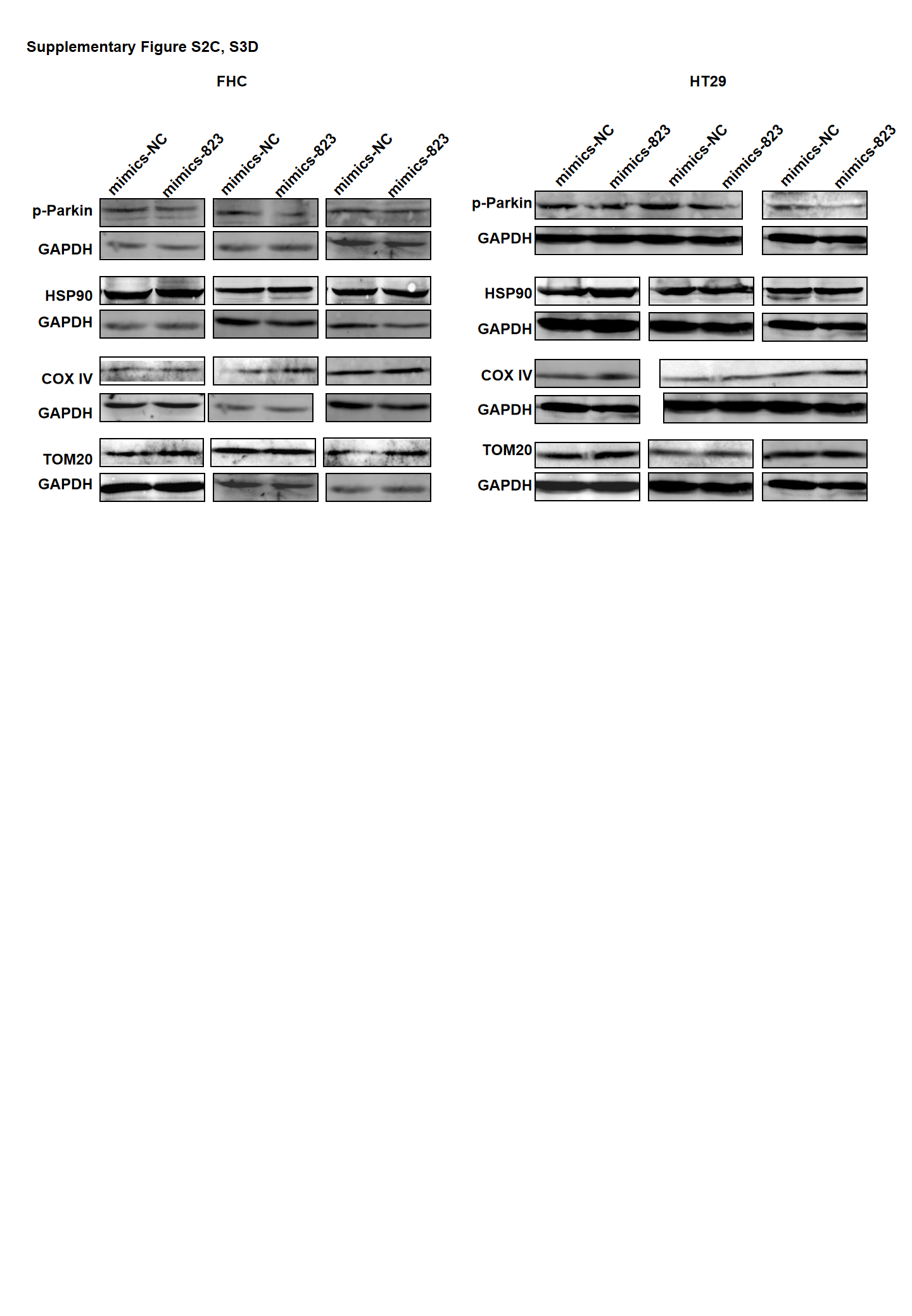

Supplement: Supplementary file 5 — WB-gel-4 [file 41419_2022_4922_MOESM5_ESM.tif]

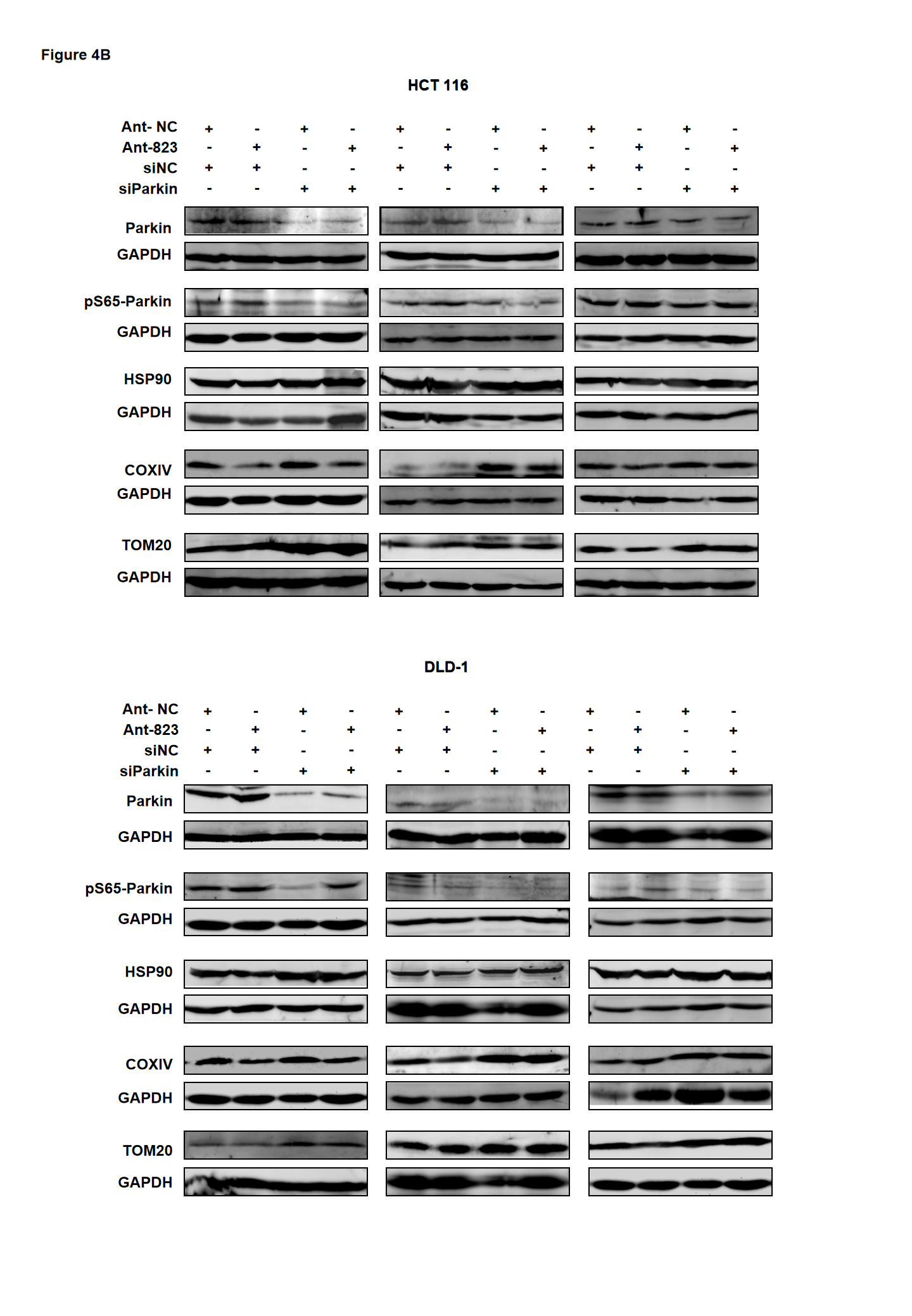

Supplement: Supplementary file 6 — WB-gel-5 [file 41419_2022_4922_MOESM6_ESM.tif]

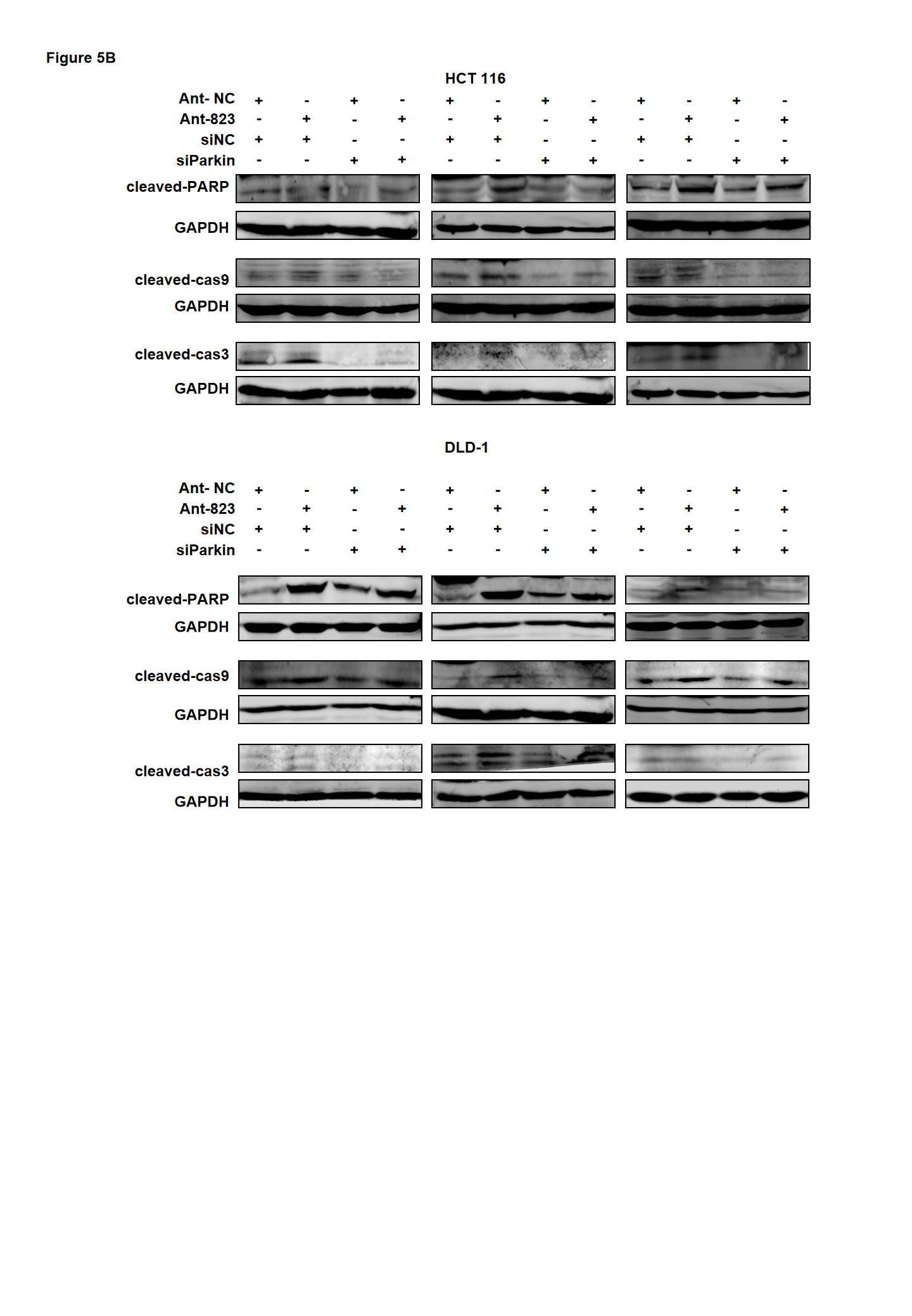

Supplement: Supplementary file 7 — WB-gel-6 [file 41419_2022_4922_MOESM7_ESM.tif]

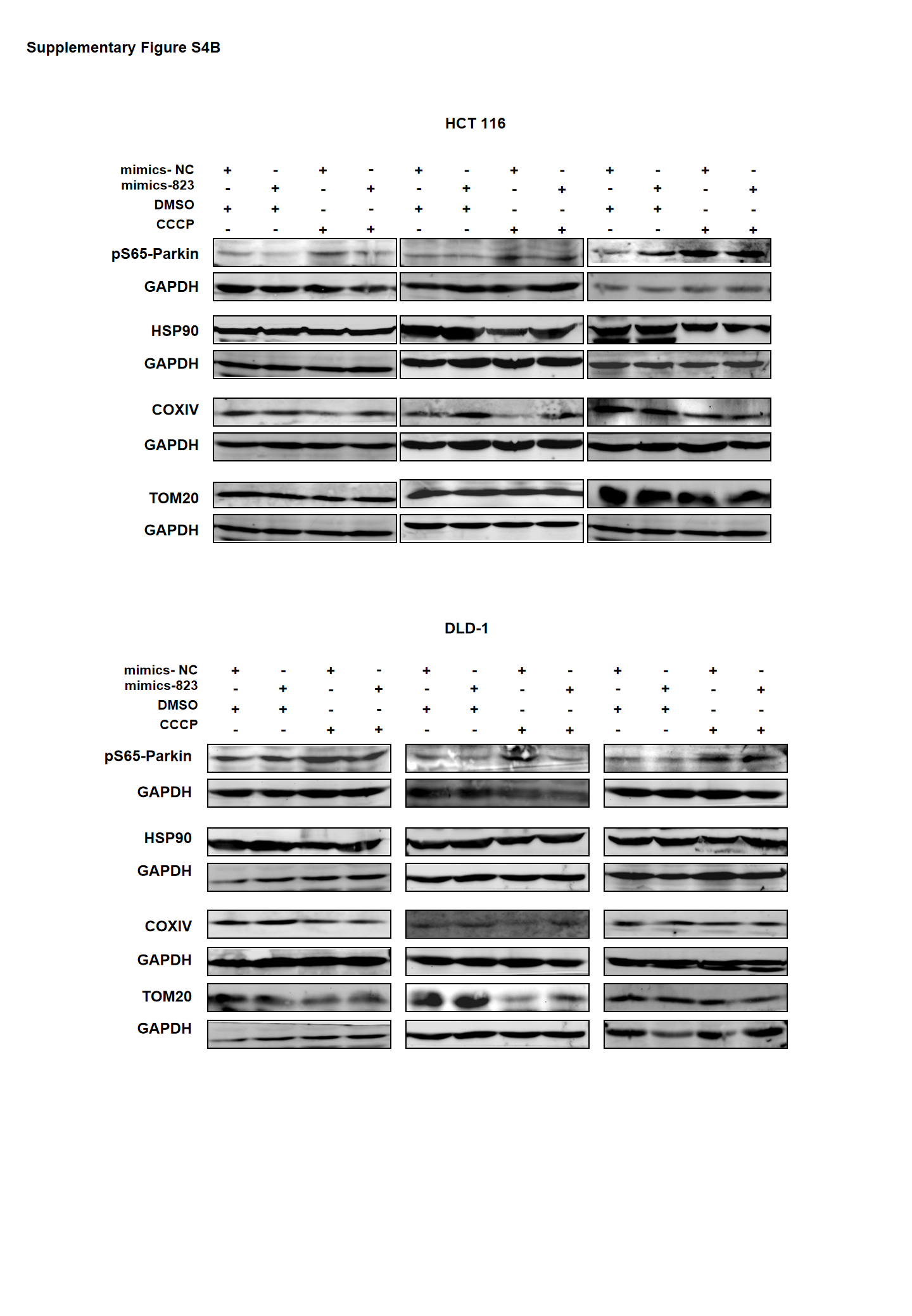

Supplement: Supplementary file 8 — WB-gel-7 [file 41419_2022_4922_MOESM8_ESM.tif]

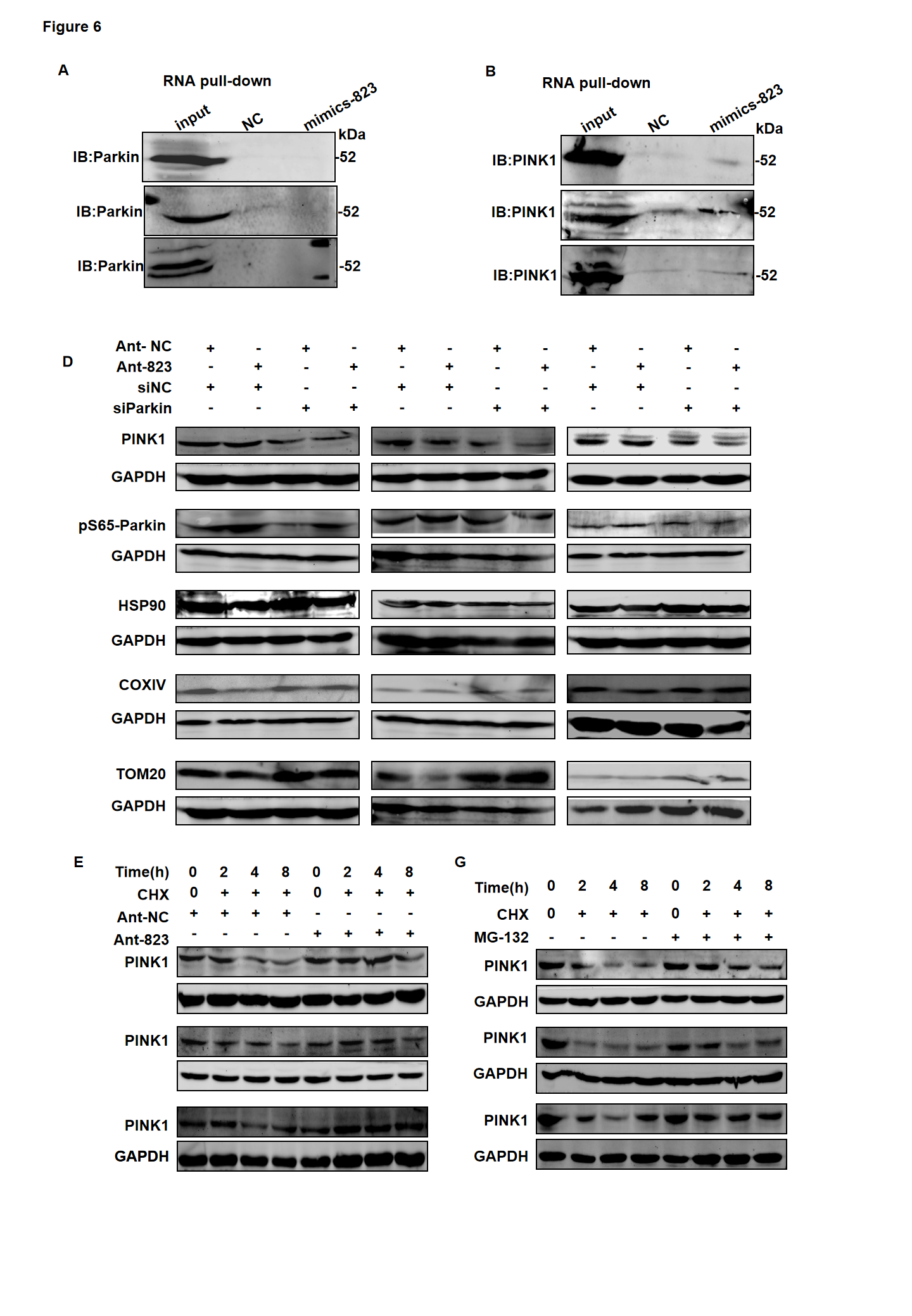

Supplement: Supplementary file 9 — WB-gel-8 [file 41419_2022_4922_MOESM9_ESM.tif]

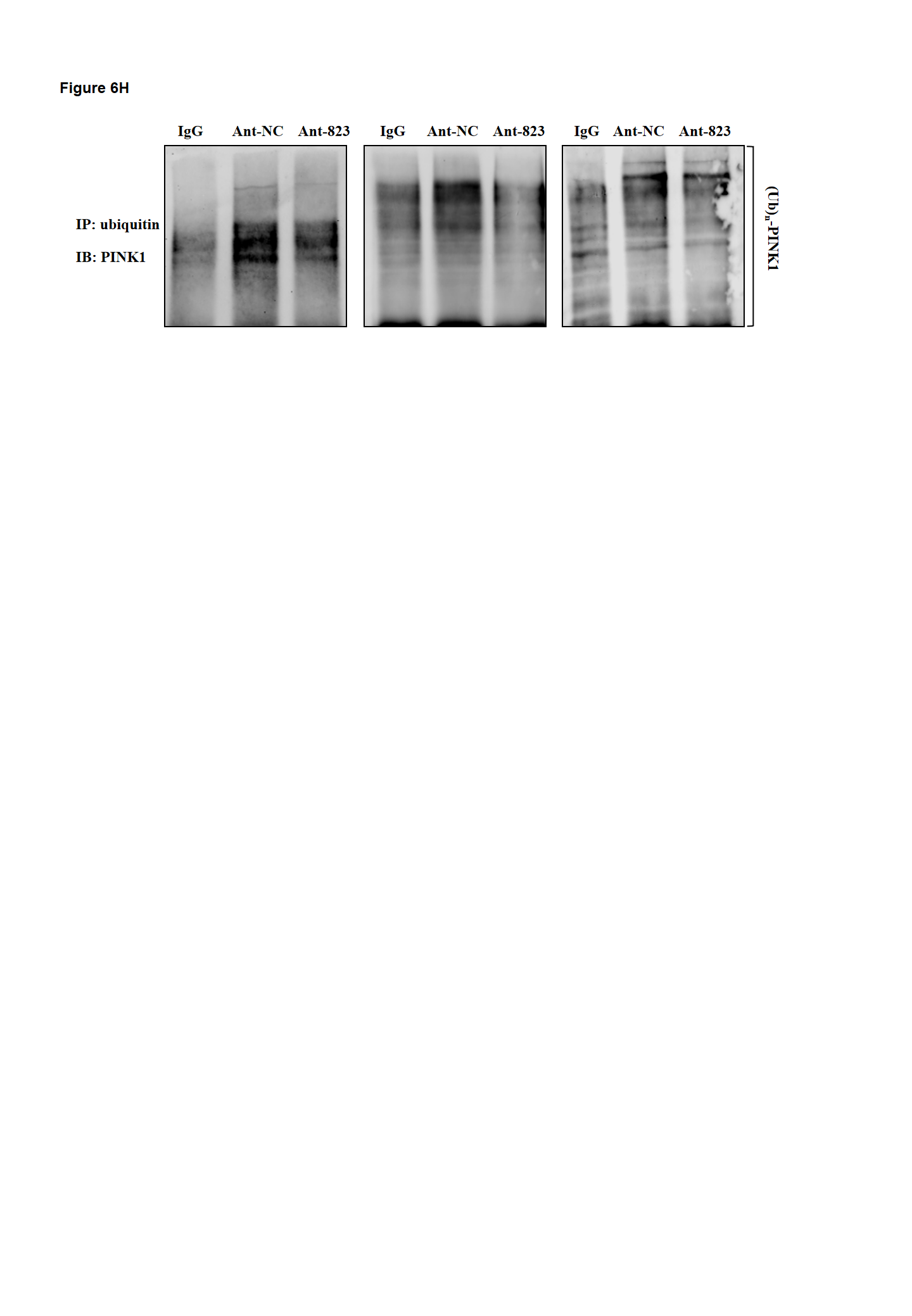

Supplement: Supplementary file 10 — WB-gel-9 [file 41419_2022_4922_MOESM10_ESM.tif]

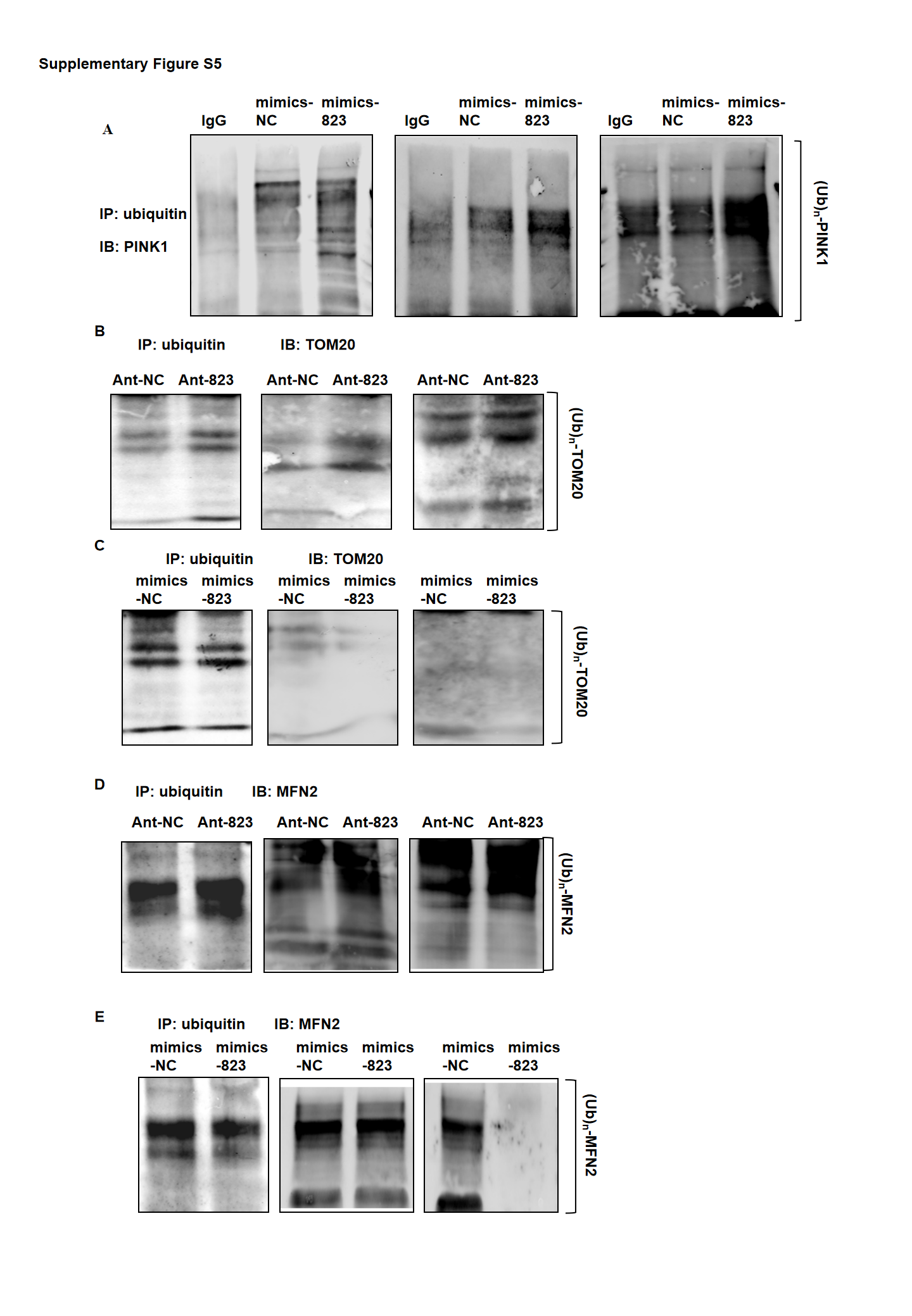

Supplement: Supplementary file 11 — WB-gel-10 [file 41419_2022_4922_MOESM11_ESM.tif]

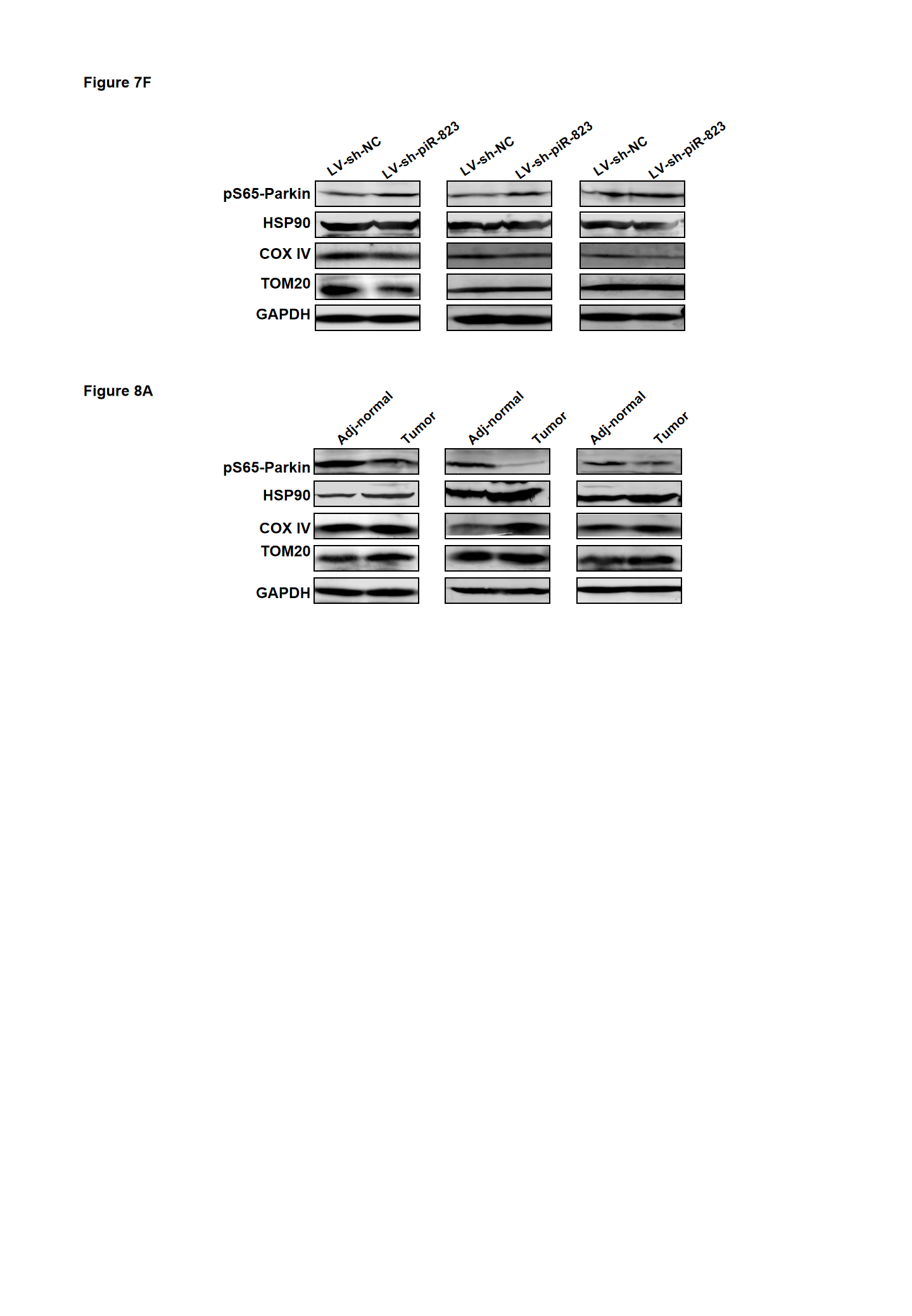

Supplement: Supplementary file 12 — WB-gel-11 [file 41419_2022_4922_MOESM12_ESM.tif]

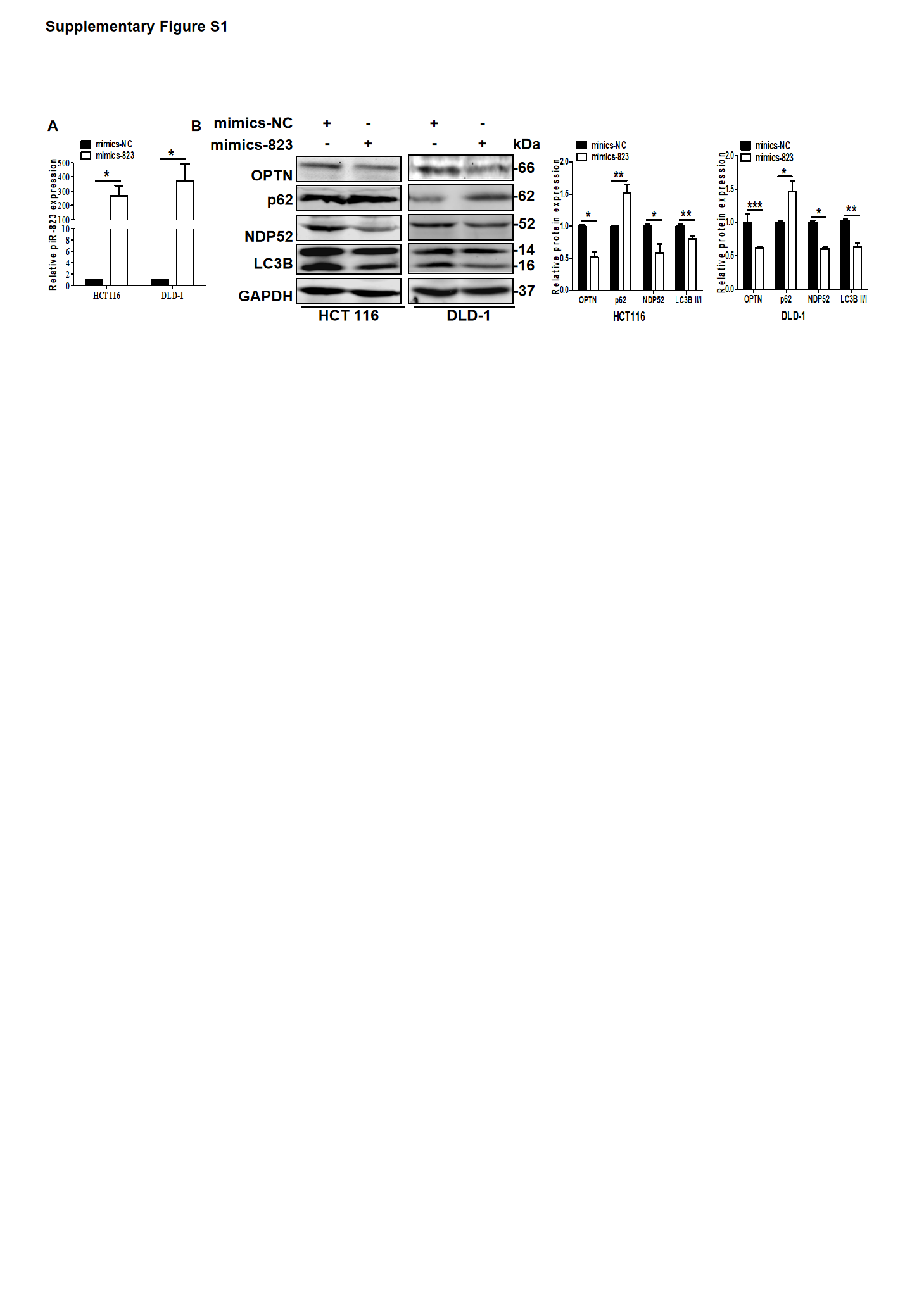

Supplement: Supplementary file 13 — Supplementary Fig. 1 [file 41419_2022_4922_MOESM13_ESM.tif]

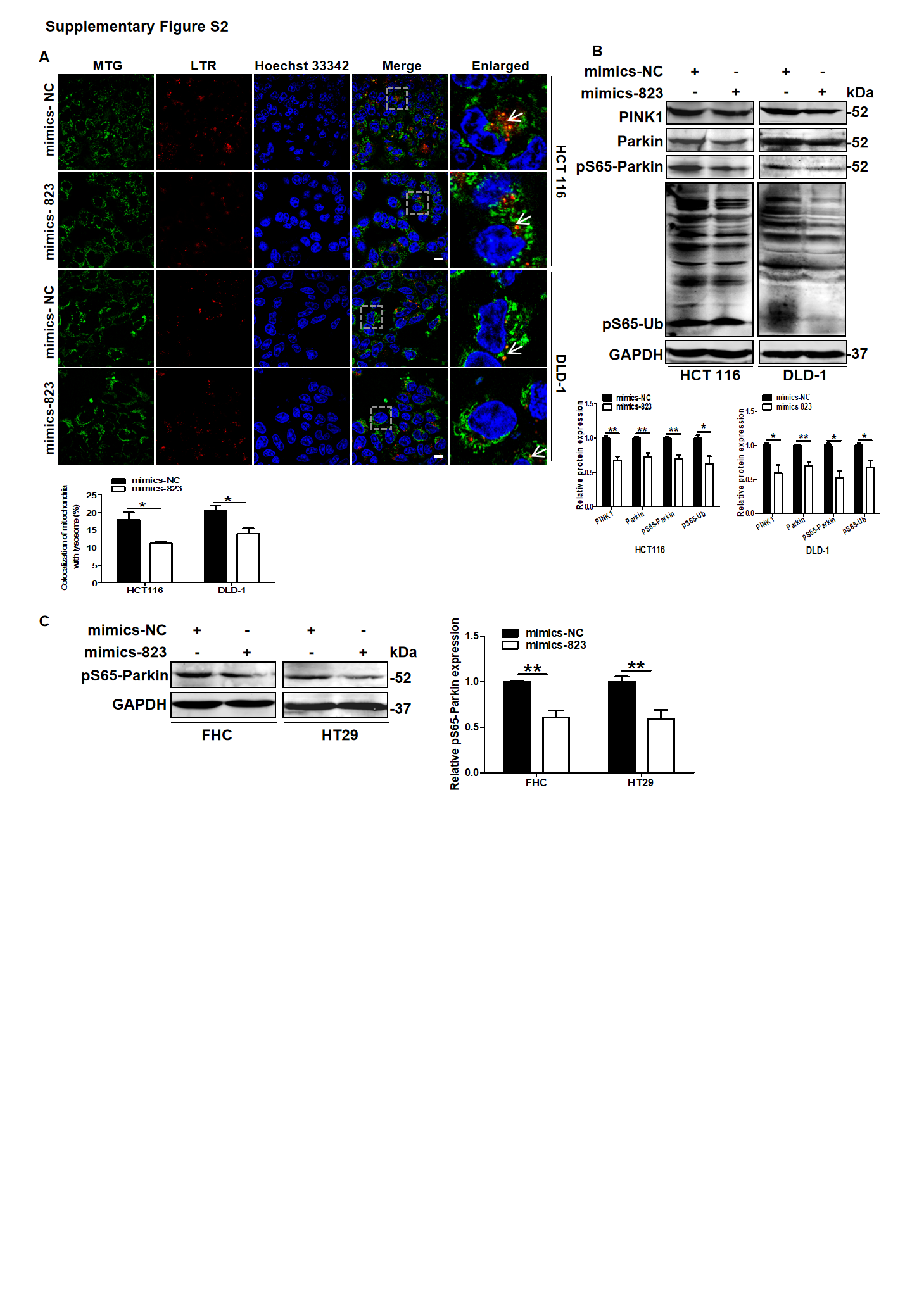

Supplement: Supplementary file 14 — Supplementary Fig. 2 [file 41419_2022_4922_MOESM14_ESM.tif]

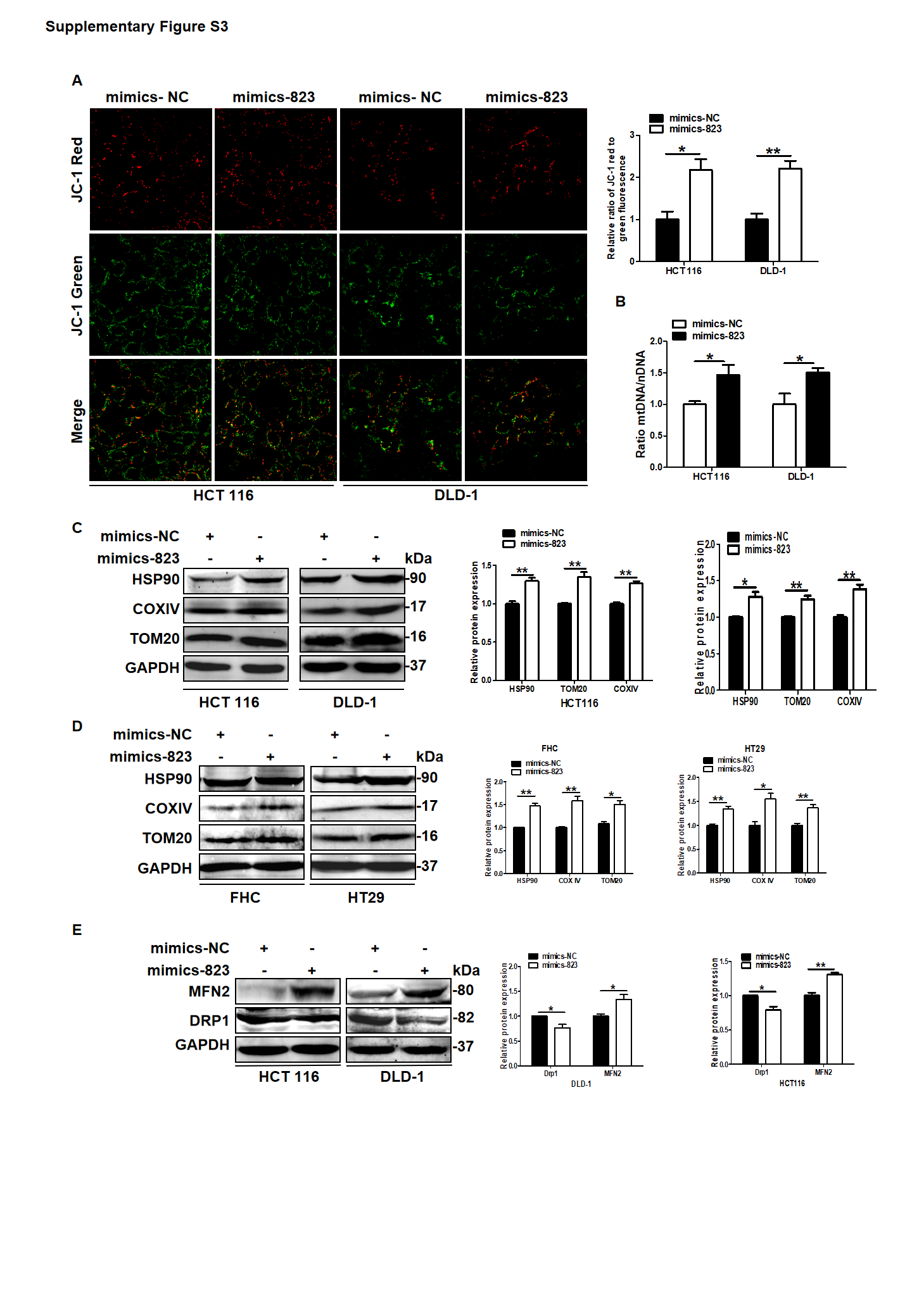

Supplement: Supplementary file 15 — Supplementary Fig. 3 [file 41419_2022_4922_MOESM15_ESM.tif]

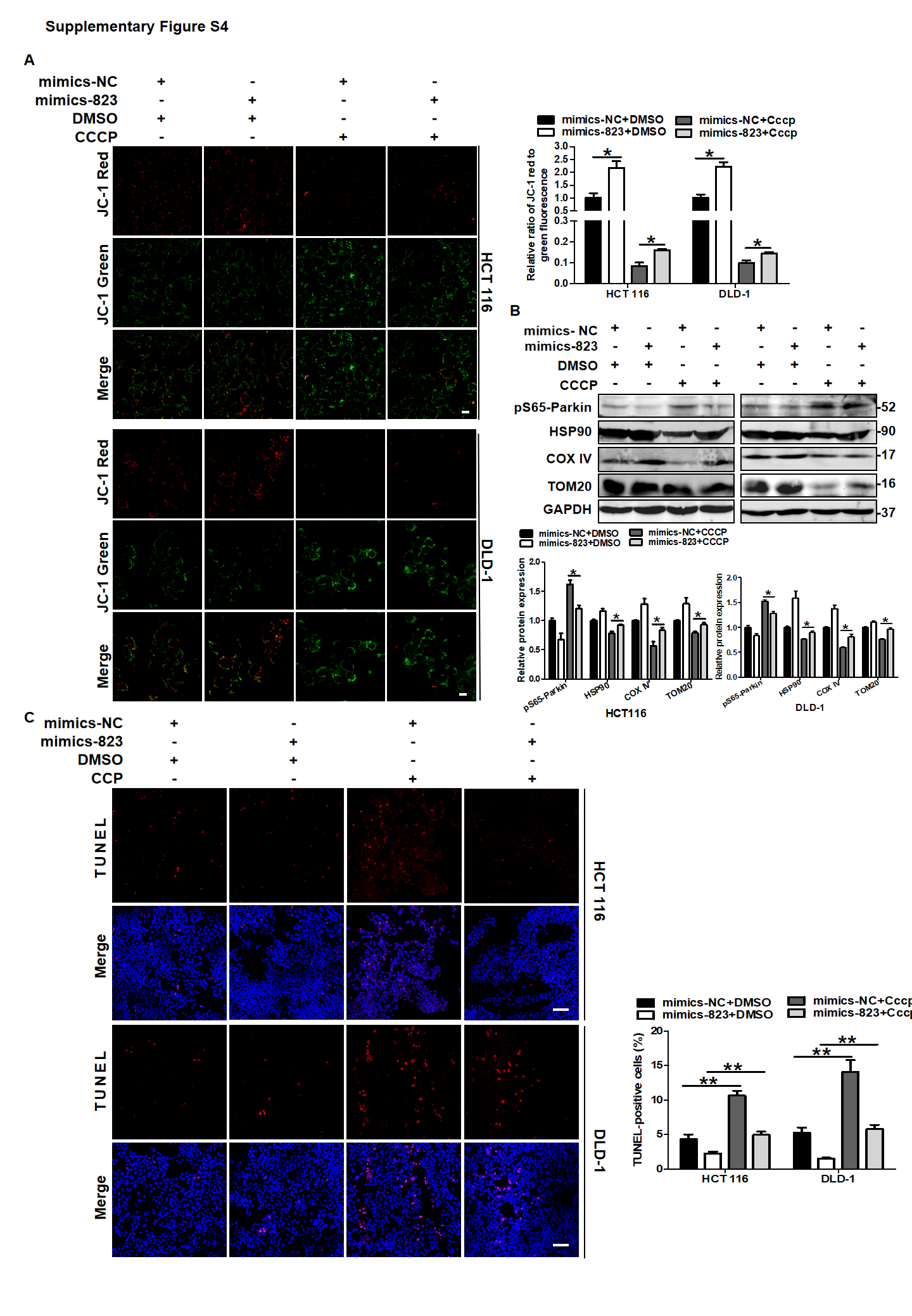

Supplement: Supplementary file 16 — Supplementary Fig. 4 [file 41419_2022_4922_MOESM16_ESM.tif]

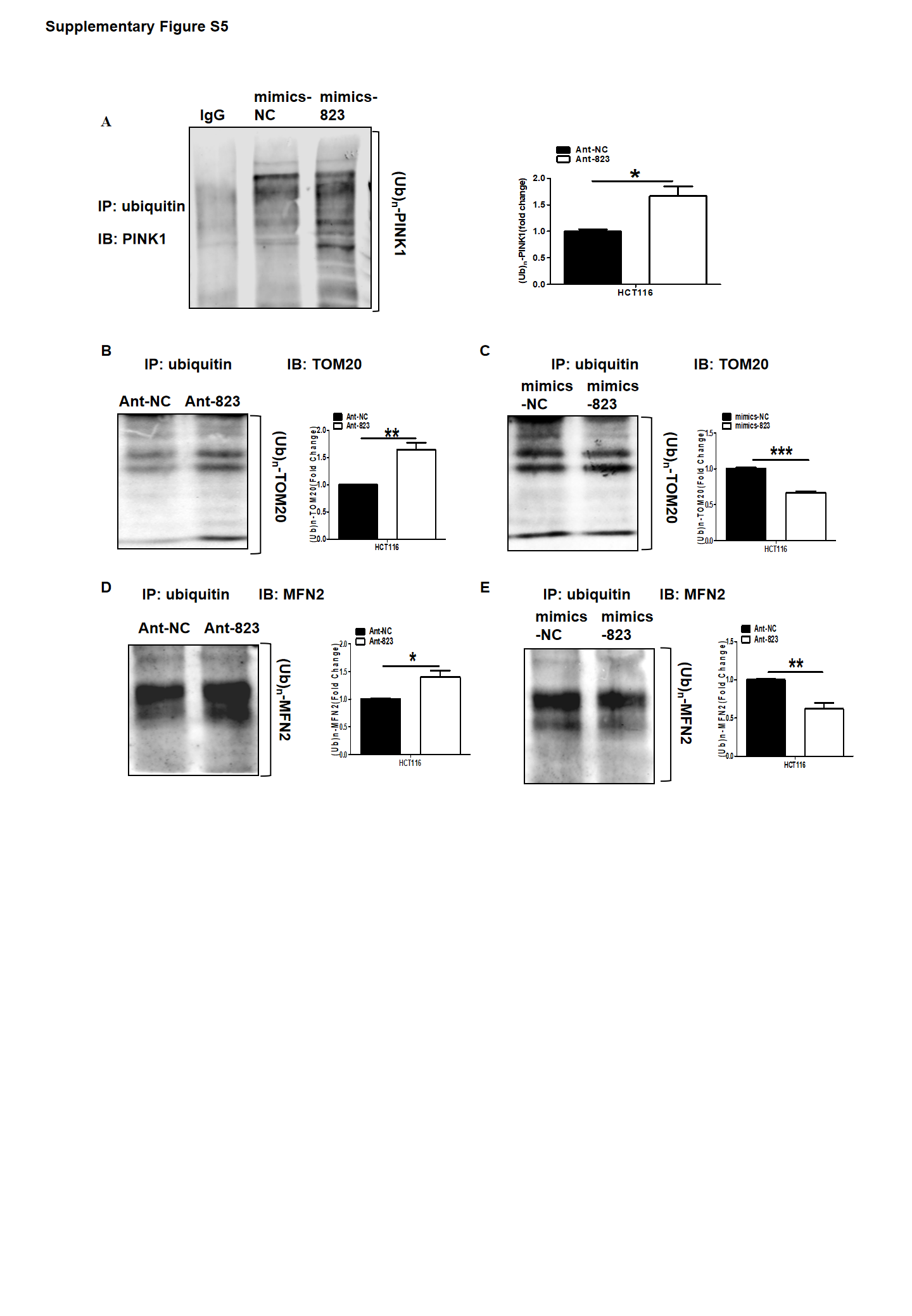

Supplement: Supplementary file 17 — Supplementary Fig. 5 [file 41419_2022_4922_MOESM17_ESM.tif]
