# Supplementary material for: piR-823 inhibits cell apoptosis via modulating mitophagy by binding to PINK1 in colorectal cancer
Source: Cell Death Dis. 2022 May 17;13(5):465. doi: 10.1038/s41419-022-04922-6 (PMC9114376; doi:10.1038/s41419-022-04922-6)
Supplement: Supplementary file 19 — Extended Data 2 [file 41419_2022_4922_MOESM19_ESM.pdf]

DECLARATION OF CONTRIBUTIONS TO ARTICLE

ADMC

Manuscript Number: CDDIS-21-2609

Journal Name: *Cell Death & Disease* (the 'Journal')

Proposed Title of the Contribution:

piR-823 inhibits cell apoptosis via modulating mitophagy by binding to PINK1 in colorectal cancer (the 'Contribution')

Author(s):

Shuling Wang, Xiaoyu Jiang, Xiaoli Xie, Jie Yin, Jiuna Zhang, Ting Liu, Shujia Chen, Yijun Wang, Xue Zhou, Yongjuan Wang, Ruolin Cui and Huiqing Jiang (the 'Authors')

For all *CDDis* articles, each person named as an author in the published version must be able to show he or she has contributed substantially to the article.

Authorship credit should be based on 1) substantial contributions to conception and design, acquisition of data, or analysis and interpretation of data; 2) drafting the article or revising it critically for important intellectual content; and 3) final approval of the version to be published. Authors should meet conditions 1, 2 and 3.

Any person who cannot be shown to have made a substantial contribution to the article cannot be listed as an author in the final version. The name of any person who is deemed to have made a minor contribution can, however, appear in the Acknowledgments section of the article.

Please complete the table below to indicate the contributions of all named authors to the manuscript.

Author Full Name:	Specification of Contribution to the Manuscript
Shuling Wang	conceived, designed the research, performed most of the experiments, treated data, and wrote the manuscript.
Huiqing Jiang	conceived, designed the research;helped to supervised the study and edited the paper.
Xiaoyu Jiang	conceived, designed the research;helped to supervised the study and edited the paper.
Xiaoli Xie	conceived, designed the research;helped to supervised the study and edited the paper; provided material and technical assistance.
Jie Yin	conceived, designed the research
Jiuna Zhang	provided material and technical assistance
Ting Liu	provided material and technical assistance
Shujia Chen	provided material and technical assistance
Yijun Wang	provided material and technical assistance
Xue Zhou	provided material and technical assistance
Yongjuan Wang	provided material and technical assistance
Ruolin Cui	provided material and technical assistance

Please complete the table below to indicate the contributions of all named authors to the figures.

Figure 1:

Shuling Wang, Xiaoyu Jiang, Xiaoli Xie, Jie Yin and Huiqing Jiang

Figure 2:

Shuling Wang, Xiaoyu Jiang, Xiaoli Xie, Jiuna Zhang, Ting Liu, Ruolin Cui and Huiqing Jiang

Figure 3:

Shuling Wang, Xiaoyu Jiang, Xiaoli Xie, Jiuna Zhang, Ting Liu, Shujia Chen and Huiqing Jiang

Figure 4:

Jie Yin, Jiuna Zhang, Ting Liu, Shujia Chen, Yijun Wang, Xue Zhou, Yongjuan Wang and Huiqing Jiang

Figure 5:

Shuling Wang, Xiaoyu Jiang, Xiaoli Xie, Jiuna Zhang, Ting Liu and Huiqing Jiang

Figure 6:

Shuling Wang, Xiaoyu Jiang, Xiaoli Xie and Huiqing Jiang

Signed for and on behalf of the Author(s):	Print Name:	Date:
Huiqing	Huiqing Jiang	2021-7-5
