# Supplementary material for: piR-823 inhibits cell apoptosis via modulating mitophagy by binding to PINK1 in colorectal cancer
Source: Cell Death Dis. 2022 May 17;13(5):465. doi: 10.1038/s41419-022-04922-6 (PMC9114376; doi:10.1038/s41419-022-04922-6)
Supplement: Supplementary file 21 — Extended Data 4 [file 41419_2022_4922_MOESM21_ESM.pdf]

细胞 STR 分型检验报告

Report of Cell Line Identification

客户名称/Applicant：	河北医科大学第二医院
样本编号/ Sample No.：	HCT116
待检测细胞系名称/Name of cell line:	HCT116
样本数量及规格/ Sample Spec.：	细胞沉淀 1 个/ Cell precipitation
样本接收日期/ Sample Receive Date：	20190812
报告编号/ Report No：	VC20190815001

1. 测试要求/Service Description

鉴定该细胞样品是否存在交叉污染现象，并与 ATCC、DSMZ 数据库比对 DNA 分型数据确认来源。

Detection of human origin intra-species cross-contamination. Database search and analysis to identify cell origin of sample using two recognized repositories (ATCC, DSMZ).

2. 检材处理和检验方法/Method and Procedure

取适量检材用莱枫痕量试剂盒提取 DNA，采用人类 STR 扩增荧光检测试剂盒进行复合 PCR 扩增，在 ABI 3730xl 型遗传分析仪上对 STR 位点和性别基因 Amelogenin 进行检测。

Cellular DNA is purified with lifefeng DNA kit. PCR is amplified with Human STR Identification Kit. PCR products are assayed with 3730xl DNA Analyzer (Applied Biosystems).

3. 检验结果/STR Profiles

该细胞株的 STR 位点和 Amelogenin 位点的基因分型结果见附表 1，分型图谱见附图 1。

The STR profiles of the cell line sample are summarized in Table 1 and Figure 1.

4. 检验结论/Result & Analysis

样本编号/Sample No.：HCT116

1) 用 GeneMapperID-X 1.4 software(ABI)对各 STR 位点进行基因型分析。该细胞 DNA 扩增后图谱清晰，分型结果良好。
   STR Typing profile is analyzed with GeneMapper ID-X 1.4 software (Applied Biosystems) (Table 1 and Figure 1).
2) 性别基因 Amelogenin：X。
3) 该株细胞 DNA 进行细胞 STR 分型结果显示（如图 1），在一个位点出现多等位基因。
   Multiple peaks were observed at one locus (Figure 1).
4) 该株细胞 DNA 分型在 ATCC & DSMZ 数据库中找到与其细胞分型89%匹配的细胞（HCT116，ATCC P/N:CCL-247，图 2,3)。
   89% matched cell line (HCT116, ATCC P/N: CCL-247) is found in ATCC & DSMZ data banks. (Figure 2,3).

审核人/Reviewed by：邓昌焕/Changhuan Deng

Shanghai VivaCell Biosciences Ltd. 上海龙田生物科技有限公司 检验专用章

表 1：样本 HCT116 的 STR 位点和 Amelogenin 位点的基因分型结果

Table 1:STR profiles of Sample

	**Sample**<br>HCT116	**Source(s): ATCC;DSMZ;**<br>HCT116, 89% Match
**Marker**	**Allele**	**Allele**
D19S433	12,13	
D5S818	10,11	10,11
D21S11	29,30	
D18S51	17	
D6S1043	13	
AMEL	X	X,Y
D3S1358	12,18,19	
D13S317	10,12	10,12
D7S820	11,12	11,12
D16S539	11,13	11,13
CSF1PO	7,10	7,10
Penta D	9,13	
D2S441	11,12	
vWA	17,22	17,22
D8S1179	12,14	
TPOX	8	8,9
Penta E	13,14	
TH01	8,9	8,9
D12S391	17,21	
D2S1338	16	
FGA	18,23	

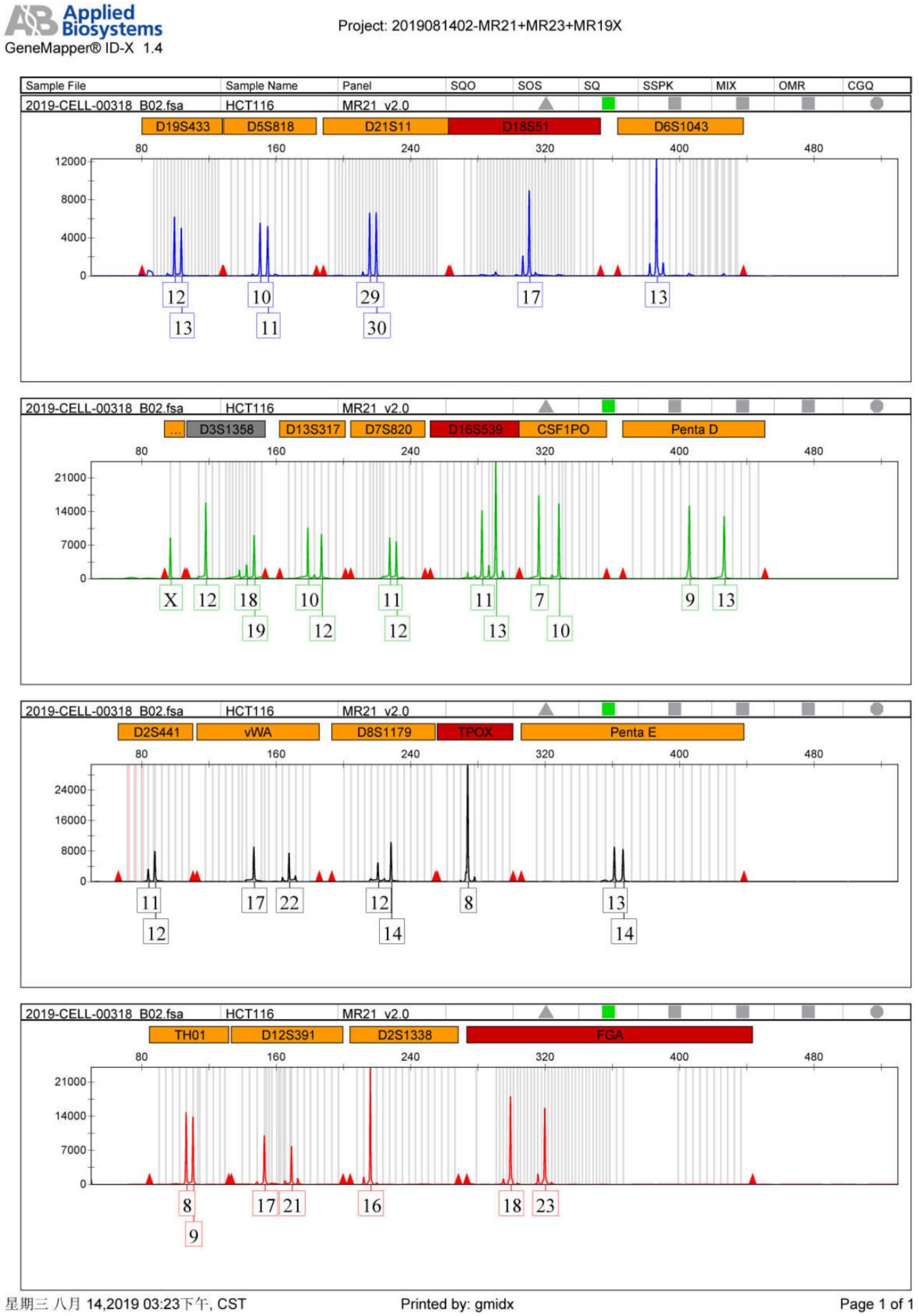

图 1/Figure 1：样本 HCT116 的 STR 位点和 Amelogenin 位点的基因分型图（STR profiles of Sample）

Add to Cart	%Match	ATCC® Number	Designation	D5S818	D13S317	D7S820	D16S539	vWA	TH01	AMEL	TPOX	CSF1PO
	89.0	CCL-247	HCT 116Colon CarcinomaHuman	10,11	10,12	11,12	11,13	17,22	8,9	X,Y	8,9	7,10
	88.0	CRL-2780	ATRFLOXColon CarcinomaHuman	10,12	10,12	11,12	11,13	17,22	8,9	X	8	7,9
	70.0	CRL-2321	HCC1143Breast CarcinomaHuman	11	12	12	11,13	16	9.3	X	12	10
	69.0	CRL-1918	CFPAC-1Pancreatic CarcinomaHuman	10,11	12	8,10	9,11	17	8	X,Y	8	10
	69.0	CRL-1997	HPAF-IIPancreas AdenocarcinomaHuman	11,13	12	10,13	11,13	17	9	X	8	10,11
	69.0	CRL-5915	NCI-H2052MesotheliomaHuman	11	12	12	11,13	17	7,9.3	X	8,11	10,11
	67.0	HTB-56	Calu-6Anaplastic CarcinomaHuman	11	11	10	13	17	9	X	8	12
	67.0	CRL-5963	NCI-BL2052B LymphoblastHuman	11	12	11,12	11,13	17	7,9.3	X,Y	8,11	10,11
	67.0	CRL-2329	HCC1500Breast Ductal CarcinomaHuman	11,13	10	11		13,16	9	X	8	
	64.0	HTB-66	RPMI-7951MelanomaHuman	11	11,12	11,12	11,12	17,19	9,9.3	X	8	12

图 2/Figure 2：样本 HCT116 与 ATCC 数据对比分析图 (Sample Comparison to the ATCC STR Profile Database)

Result of STR matching analysis by your data.

- DSMZ Profile Database -

A graphical presentation is shown at the bottom of this page.

EV	Cell No.	Cell name	Locus names									Figures
			D5S818	D13S317	D7S820	D16S539	VWA	TH01	AM	TPOX	CSF1PO	
	Query (Your Cell)		*10,11*	*10,12*	*11,12*	*11,13*	*17,22*	*8,9*	*X,X*	*8,8*	*7,10*	
0.89(32/36)	CCL-247	HCT 116	10,11	10,12	11,12	11,13	17,22	8,9	X,Y	8,9	7,10	-
0.89(32/36)	CRL-2780	ATRFLOX [Mutatect]	10,12	10,12	11,12	11,13	17,22	8,9	X,X	8,8	7,9	-
0.84(32/38)	581	HCT-116	10,11	10,12	11,12	11,13,12	17,17,21	8,8	X,X	8,8	7,10	-
0.67(24/36)	CRL-7266	Hs 389(B).Lu	11,12	9,12	9,11	11,13	17,18	8,9	X,X	8,8	11,11	-
0.67(24/36)	JCRB0101	A3/KAW	10,11	10,12	11,12	10,10	17,17	7,9	X,X	8,8	11,12	-
0.67(24/36)	JCRB0816	SBC-1	9,11	12,12	11,12	9,9	14,17	8,9	X,X	8,8	10,12	-
0.67(24/36)	JCRB0817	SBC-2	9,11	12,12	11,12	9,9	14,17	8,9	X,X	8,8	10,12	-
0.67(24/36)	RCB1433	TEN	11,12	10,12	10,12	11,12	14,17	7,8	X,X	8,8	10,12	-
0.61(22/36)	223	HUP-T4	10,11	8,8	8,11	10,11	17,18	9,9	X,X	8,8	10,12	-
0.61(22/36)	307	DV-90	11,13	10,12	11,14	11,12	14,15	7,8	X,X	8,8	10,12	-

图 3/ Figure 3：样本 HCT116 与 DSMZ 数据对比分析图（Sample Comparison to the DSMZ STR Profile Database）
