# Supplementary material for: piR-823 inhibits cell apoptosis via modulating mitophagy by binding to PINK1 in colorectal cancer
Source: Cell Death Dis. 2022 May 17;13(5):465. doi: 10.1038/s41419-022-04922-6 (PMC9114376; doi:10.1038/s41419-022-04922-6)
Supplement: Supplementary file 22 — Extended Data 5 [file 41419_2022_4922_MOESM22_ESM.pdf]

细胞 STR 分型检验报告

Report of Cell Line Identification

客户名称/ Applicant： 河北医科大学第二医院
样本编号/ Sample No.： HT29
待检测细胞系名称/Name of cell line: HT29
样本数量及规格/ Sample Spec.： 细胞沉淀 1 个/ Cell precipitation
样本接收日期/ Sample Receive Date： 20191025
报告编号/ Report No： VC20191029003

1. **测试要求/**Service Description

鉴定该细胞样品是否存在交叉污染现象，并与 ATCC、DSMZ 数据库比对 DNA 分型数据确认来源。

Detection of human origin intra-species cross-contamination. Database search and analysis to identify cell origin of sample using two recognized repositories (ATCC, DSMZ).

2. **检材处理和检验方法/**Method and Procedure

取适量检材用莱枫痕量试剂盒提取 DNA，采用人类 STR 扩增荧光检测试剂盒进行复合 PCR 扩增，在 ABI 3730xl 型遗传分析仪上对 STR 位点和性别基因 Amelogenin 进行检测。

Cellular DNA is purified with lifefeng DNA kit. PCR is amplified with Human STR Identification Kit. PCR products are assayed with 3730xl DNA Analyzer (Applied Biosystems).

3. **检验结果/**STR Profiles

该细胞株的 STR 位点和 Amelogenin 位点的基因分型结果见附表 1，分型图谱见附图 1。

The STR profiles of the cell line sample are summarized in Table 1 and Figure 1.

4. **检验结论/**Result & Analysis

样本编号/Sample No.：HT29

1) 用 GeneMapperID-X 1.4 software(ABI)对各 STR 位点进行基因型分析。该细胞 DNA 扩增后图谱清晰，分型结果良好。
   STR Typing profile is analyzed with GeneMapper ID-X 1.4 software (Applied Biosystems) (Table 1 and Figure 1).
2) 性别基因 Amelogenin：X。
3) 该株细胞 DNA 进行细胞 STR 分型结果显示（如图 1），未出现多等位基因，未发现明显的细胞交叉污染现象。
   Normal peaks distribution was observed (Figure 1). There was no cross-contamination of human cell lines.
4) 该株细胞 DNA 分型在 ATCC & DSMZ 数据库中找到与其细胞分型完全匹配的细胞。该细胞样本名称：HT-29, ATCC P/N: HTB-38 等（图 2,3）。
   100% matched cell line is found in ATCC & DSMZ data banks. The name of the cell line: HT-29, ATCC P/N: HTB-38 etc. (Figure 2,3)

VivaCell Biosciences 上海宠田生物科技有限公司 检验专用章

审核人/Reviewed by： 邓昌焕/Changhuan Deng

表 1：样本 HT29 的 STR 位点和 Amelogenin 位点的基因分型结果

Table 1:STR profiles of Sample

	**Sample**<br>HT29	**Source: ATCC;DSMZ;**<br>HT-29,100% Match
**Marker**	**Allele**	**Allele**
D3S1358	15,17	
D5S818	11,12	11,12
D2S1338	19,23	
TPOX	8,9	8,9
CSF1PO	11,12	11,12
Penta D	11,13	
Indel		
AMEL	X	X
TH01	6,9	6,9
vWA	17,19	17,19
D7S820	10	10
D21S11	29,30	
Penta E	14,16	
D10S1248	14	
D8S1179	10	
D1S1656	15,16	
D18S51	13	
D12S391	18.3,21	
D6S1043	12,14	
D19S433	14	
D16S539	11,12	11,12
D13S317	11,12	11,12
FGA	20,22	

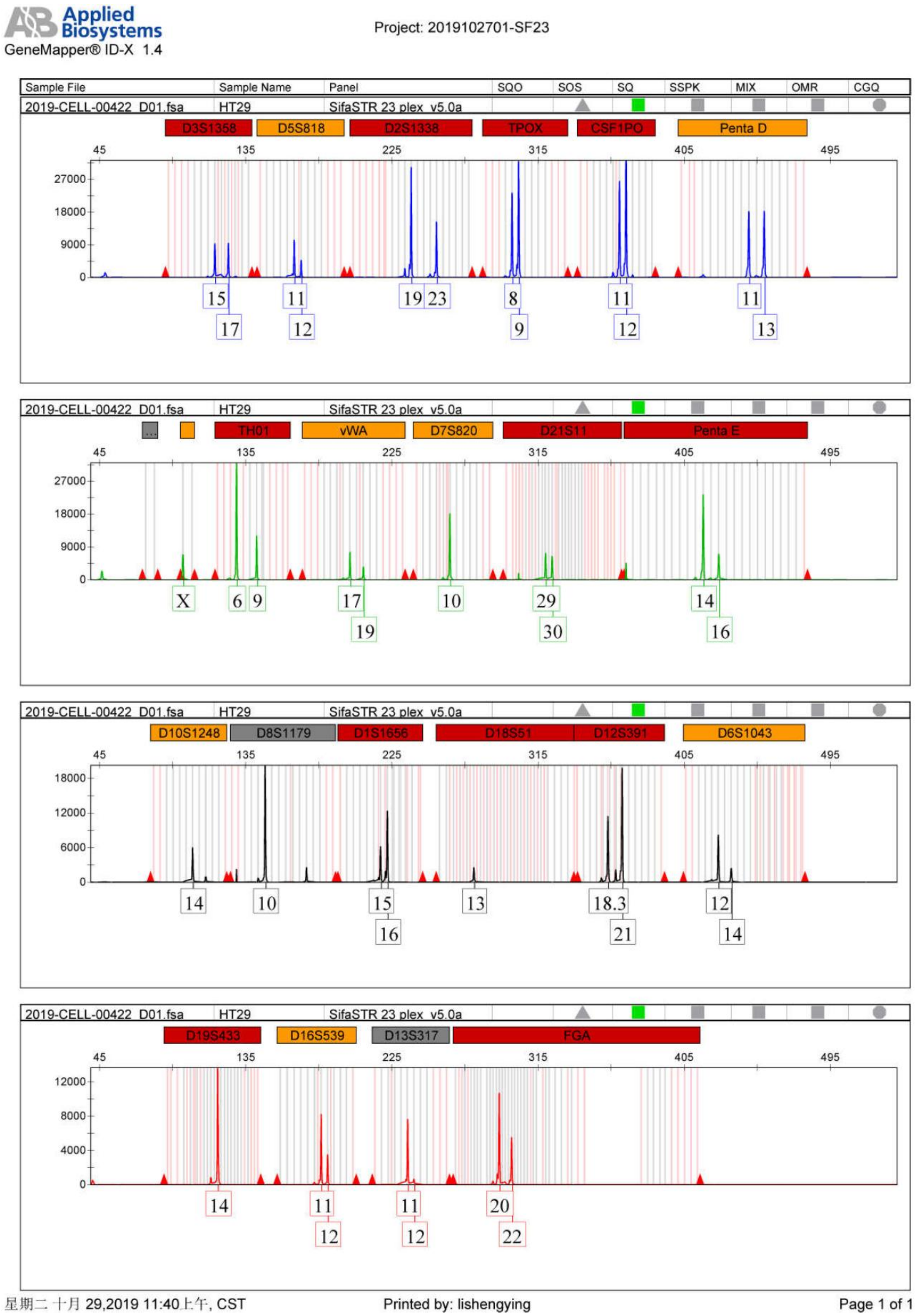

图 1/Figure 1：样本 HT29 的 STR 位点和 Amelogenin 位点的基因分型图（STR profiles of Sample）

Add to Cart	%Match	ATCC® Number	Designation	D5S818	D13S317	D7S820	D16S539	vWA	TH01	AMEL	TPOX	CSF1PO
☐	100.0	CCL-218	WiDrColon AdenocarcinomaHuman	11,12	11,12	10	11,12	17,19	6,9	X	8,9	11,12
☐	100.0	HTB-38	HT-29Colon AdenocarcinomaHuman	11,12	11,12	10	11,12	17,19	6,9	X	8,9	11,12
☐	92.0	HTB-161	NIH:OVCAR-3Ovarian AdenocarcinomaHuman	11,12	12	10	12	17	9,9.3	X	8	11,12
☐	89.0	HTB-56	Calu-6Anaplastic CarcinomaHuman	11	11	10	13	17	9	X	8	12
☐	83.0	CRL-5902	NCI-H1876Lung CarcinomaHuman	11	12	10,12	12	17	6	X,Y	8,9	11
☐	83.0	CRL-5903	NCI-H1882Lung CarcinomaHuman	11	12	10,12	12	17	6	X,Y	8,9	11
☐	82.0	HTB-35	SiHaCervical CarcinomaHuman	9	11	10	12	14,17	6,9	X	8	12
☐	79.0	HTB-66	RPMI-7951MelanomaHuman	11	11,12	11,12	11,12	17,19	9,9.3	X	8	12
☐	79.0	CRL-5808	NCI-H378Small Cell Lung CancerHuman	11,12	11	12	12	16,19	6,9.3	X	8,9	11,12
☐	77.0	CRL-10302	SW756Cervical CarcinomaHuman	11,12	11	10,12	12,13	17	9.3	X	8	11,12
☐	77.0	CRL-2553	Panc 02.03Pancreas AdenocarcinomaHuman	12,13	12	9,10	11	17	6	X	9,12	11,12

图 2/Figure 2：样本 HT29 与 ATCC 数据对比分析图 (Sample Comparison to the ATCC STR Profile Database)

Result of STR matching analysis by your data.

- DSMZ Profile Database -

A graphical presentation is shown at the bottom of this page.

EV	Cell No.	Cell name	D5S818	D13S317	D7S820	D16S539	VWA	TH01	AM	TPOX	CSF1PO	Figures
	Query (Your Cell)		*11,12*	*11,12*	*10,10*	*11,12*	*17,19*	*6,9*	*X,X*	*8,9*	*11,12*	
1.00(36/36)	299	HT-29	11,12	11,12	10,10	11,12	17,19	6,9	X,X	8,9	11,12	-
1.00(36/36)	CCL-218	WiDr	11,12	11,12	10,10	11,12	17,19	6,9	X,X	8,9	11,12	-
1.00(36/36)	CRL-2519	MV522	11,12	11,12	10,10	11,12	17,19	6,9	X,X	8,9	11,12	-
1.00(36/36)	CRL-2520	MV522/MDR1	11,12	11,12	10,10	11,12	17,19	6,9	X,X	8,9	11,12	-
1.00(36/36)	CRL-3026	USC-HNI	11,12	11,12	10,10	11,12	17,19	6,9	X,X	8,9	11,12	-
1.00(36/36)	HTB-38	HT-29	11,12	11,12	10,10	11,12	17,19	6,9	X,X	8,9	11,12	-
1.00(36/36)	IFO50043	WiDr	11,12	11,12	10,10	11,12	17,19	6,9	X,X	8,9	11,12	-
1.00(36/36)	JCRB0224	WiDr	11,12	11,12	10,10	11,12	17,19	6,9	X,X	8,9	11,12	-
0.94(34/36)	129	CX-1	11,12	11,11	10,10	11,12	17,19	6,9	X,X	8,9	11,12	-
0.89(32/36)	CRL-2521	MV522/MRP	11,12	11,12	10,10	11,12	17,19	6,6	X,X	8,9	12,12	-

图 3/ Figure 3：样本 HT29 与 DSMZ 数据对比分析图（Sample Comparison to the DSMZ STR Profile Database）
