# Supplementary material for: piR-823 inhibits cell apoptosis via modulating mitophagy by binding to PINK1 in colorectal cancer
Source: Cell Death Dis. 2022 May 17;13(5):465. doi: 10.1038/s41419-022-04922-6 (PMC9114376; doi:10.1038/s41419-022-04922-6)
Supplement: Supplementary file 23 — Extended Data 6 [file 41419_2022_4922_MOESM23_ESM.pdf]

SPRINGER NATURE

Author Services

Editing Certificate

This document certifies that the manuscript

piR-823 inhibits colon cancer cell apoptosis by modulating mitophagy

prepared by the authors

Shu-Ling Wang, Xiao-Yu Jiang, Xiao-Li Xie, Jie Yin, Jiu-Na Zhang, Ting Liu, Shu-Jia Chen, Yi-Jun Wang, Xue Zhou, Yong-Juan Wang, Ruo-Lin Cui...

was edited for proper English language, grammar, punctuation, spelling, and overall style by one or more of the highly qualified native English speaking editors at SNAS.

This certificate was issued on **January 27, 2021** and may be verified on the SNAS website using the verification code **884A-5A37-D009-D560-DC71**.

Neither the research content nor the authors' intentions were altered in any way during the editing process. Documents receiving this certification should be English-ready for publication; however, the author has the ability to accept or reject our suggestions and changes. To verify the final SNAS edited version, please visit our verification page at secure.authorservices.springernature.com/certificate/verify.
If you have any questions or concerns about this edited document, please contact SNAS at support@as.springernature.com.

SNAS provides a range of editing, translation, and manuscript services for researchers and publishers around the world.
For more information about our company, services, and partner discounts, please visit authorservices.springernature.com.
